# Influence of Epoch Length and Recording Site on the Relationship Between Tri-Axial Accelerometry-Derived Physical Activity Levels and Structural, Functional, and Hemodynamic Properties of Central and Peripheral Arteries

**DOI:** 10.3389/fspor.2022.799659

**Published:** 2022-02-24

**Authors:** Mariana Gómez-García, Juan Torrado, Daniel Bia, Yanina Zócalo

**Affiliations:** ^1^Departamento de Educación Física y Salud, Instituto Superior de Educación Física, Universidad de la República, Montevideo, Uruguay; ^2^Grupo “Centro Universitario de Investigación, Innovación y Diagnóstico Arterial – Movimiento, Actividad, Salud” (CUiiDARTE-MAS), Comisión Sectorial de Investigación Científica (CSIC), Universidad de la República, Montevideo, Uruguay; ^3^Department of Internal Medicine, Jacobi Medical Center, Albert Einstein College of Medicine, New York, NY, United States; ^4^Departamento de Fisiología, Facultad de Medicina, Centro Universitario de Investigación, Innovación y Diagnóstico Arterial (CUiiDARTE), Universidad de la República, Montevideo, Uruguay

**Keywords:** accelerometry, arterial system, cardiovascular, epoch lengths, hip, physical activity, recording site, worn on wrist option (ActiLife software)

## Abstract

**Background:**

It remains to be established to what extent physical activity (PA) levels among individuals are independently associated with deviations from the “optimal” state of the arterial system. Accelerometers have been proposed as means to obtain reliable, objective, and more comprehensive data of PA. Decisions at the time of data collection/processing could influence the association between accelerometry-derived indices and arterial properties.

**Objectives:**

(i) To identify to what extent the strength of association between arterial properties and accelerometer-derived indices depend on the recording site and/or the epoch length; (ii) to determine whether some arterial characteristics (hemodynamic vs. structural vs. functional) or regions (elastic vs. transitional vs. muscular arteries; central vs. peripheral) have higher levels of association with accelerometry-derived indices.

**Methods:**

Physical activity (PA), cardiovascular risk factors (CRFs), and cardiovascular properties were evaluated in 60 volunteers (general population; age: 23–62 years; women: 43%). PA was measured daily for 7 days (free-living situation; triaxial-accelerometers ActiGraph-GT3X+; hip and wrist; “Worn-to-wrist” option) and raw data was converted at epoch lengths of 1, 5, 10, 30, and 60-s. PA-related energy expenditure, daily time in moderate-to-vigorous PA, steps/minute, and counts-per-minute for vector magnitude were calculated. The cardiovascular evaluation included hemodynamic (central and peripheral pressure), structural (diameters and intima-media thickness), and functional (local and regional stiffness) parameters of carotids, femoral, and brachial arteries, and carotid-femoral and carotid-radial pathways. Arterial z-scores were obtained using age-related equations derived from healthy participants not exposed to CRFs (*n* = 1,688; age: 2–84 years; female: 51.2%) to evaluate at which degree each parameter deviates from the “optimal” value.

**Methods:**

In general, hip recordings outperformed those obtained on the wrist regarding the strength of association with arterial parameters. Accelerometer-derived indices and their association with arterial properties vary depending on the recording site and epoch length. PA indices are stronger associated with functional (local) than structural variables and with central than peripheral arteries.

**Conclusions:**

Regardless of the PA index, there were independent associations with central artery characteristics, which reinforces that these territories would be the most related to PA levels. Differences in data acquisition and processing could lead to differences in conclusions when addressing the association between accelerometer-derived indices and the cardiovascular system.

## Introduction

Physical activity (PA) is associated with a reduced burden of atherosclerotic cardiovascular disease (CVD). However, it is to note that many of the studies on this topic have shown limitations related to objectively assessing the participants' PA volume (Bertoni et al., 2009). Accelerometers have emerged as useful (now widely used) tools to assess PA as they can provide practical, accurate, and reliable data, allowing ambulatory assessment of usual daily activities (John and Freedson, 2012; O'Driscoll et al., 2020). One of the strengths of the use of accelerometers is that it provides objective and quantitative participant-specific information about PA, critical to a better understanding of the biological links between human activity behavior and the cardiovascular and/or metabolic status (Aadland et al., 2018, 2020). However, methodological issues related to the use of accelerometers remain under debate. In particular, raw data obtained and delivered by the accelerometer is extensive, complex, and subjected to noise, artifacts, and sampling-related issues—reasons why researchers have proposed different data processing and analysis decisions or approaches to simplify the information at the time of creating accelerometer-derived PA estimates (John and Freedson, 2012; Aibar and Chanal, 2015; Banda et al., 2016; Kerr et al., 2016, 2017). Not surprisingly, the above resulted in differences when analyzing accelerometer-derived signals and determining PA (levels and patterns) in different populations (Edwardson and Gorely, 2010; Gabriel et al., 2010; Ojiambo et al., 2011; Sanders et al., 2014; Aibar and Chanal, 2015; Kerr et al., 2016, 2017). The extent to which the methodological approach could impact the capacity of accelerometer-derived indices to predict cardiovascular status is incompletely understood.

One key decision is to define the “epoch length” or “sampling interval” (e.g., 1, 5, 10, 30, 60 s), which refers to the interval of time over which the units of accelerometer measures (“counts”) are summed (Aibar and Chanal, 2015; Banda et al., 2016). Previous studies showed that the epoch lengths have a significant impact on the classification of sedentary and moderate-to-vigorous PA (MVPA) time and the observed compliance to MVPA guidelines (Edwardson and Gorely, 2010; Gabriel et al., 2010; Ojiambo et al., 2011; Sanders et al., 2014; Aibar and Chanal, 2015; Quante et al., 2015; Nettlefold et al., 2016; Aadland et al., 2018, 2020). The above would be the result of changes in PA distribution across different intensity levels when different epoch lengths are used (Aibar and Chanal, 2015; Aadland et al., 2018, 2020). In free-living conditions, a “short epoch” is strongly recommended to obtain a “real picture” of PA behavior in children and adolescents as it would avoid the accumulation of counts reflecting the average activity (“smoothing effect”) expected when long epochs are used (Edwardson and Gorely, 2010; Aadland et al., 2018, 2020). The above is not in agreement with some authors “findings” (Sanders et al., 2014; Aibar and Chanal, 2015). In addition, the “ideal epoch length” could differ depending on the objective pursued. Altenburg et al. (2021) concluded that whereas 60-s epochs would be of choice to classify sedentary behaviors, shorter epoch lengths would be necessary to capture short bursts of MVPA. Furthermore, the “epoch length” could determine the association between PA levels and markers of the metabolic status as was previously proposed (Aadland et al., 2018, 2020). About this, for data analyzed using the 60-s epoch, moderate PA intensities and metabolic health markers were associated, but the associations weakened when shorter epoch lengths were considered (Aadland et al., 2020). In this context, it is to note that it remains unknown whether the epoch length impacts the association between accelerometer-derived PA indices and the hemodynamic, structural, or functional arterial properties.

Another important definition is the accelerometer location (e.g., hip, wrist, thigh, ankle, neck). Although a hip-mounted accelerometer has been traditionally used to measure PA, the use of wrist-worn devices has gained popularity (Troiano et al., 2008). Wrist-accelerometer is easy to carry and can be worn for 24 h without removal for sleep, which may improve compliance with wearing patterns (Migueles et al., 2017). However, signals derived from wrist accelerometers could be affected by accelerations related to arm movements not necessarily associated with higher PA-related energy expenditure (EE) (Hildebrand et al., 2014; Ellis et al., 2016). This has led some devices to implement conversion algorithms (equations or filters) that attempt to standardize the levels of accelerometer-derived PA indices (e.g., EE levels), regardless of the recording site (e.g., hip or wrist; McMinn et al., 2013; Mandigout et al., 2019; Mueller et al., 2020; Nuss et al., 2020; ActiGraph, 2021). However, as mentioned for epoch lengths, it remains to be defined whether the accelerometer position (even applying data correction schemes to make different recording sites comparable) determines the strength of the association between PA indices and the arterial status.

Finally, a third decision is the selection of the most appropriate accelerometer-derived PA index to be used. Data processing from accelerometry usually provides a large number of indices, which may not necessarily be related to the arterial state. In this regard, when analyzing the association between PA and the cardiovascular system, the accelerometer-derived index to be selected would be the one with the greatest predictor capacity for cardiovascular status. In addition, it is important to determine whether a given PA index is mainly associated with (i) hemodynamic [e.g., central or peripheral blood pressure (BP)], structural (e.g., diameter, wall thickness), or functional (e.g., stiffness) characteristics, or with the status of (ii) a particular histological arterial type [e.g., elastic (carotid), muscular (femoral), or transitional (brachial)] (Bia et al., 2009). In previous studies, we found that the impact of different cardiovascular risk factors (CRFs) could differ depending on the vascular parameter and territory considered (Zócalo et al., 2017; Zocalo et al., 2018; Garcia-Espinosa et al., 2018; Castro et al., 2019, 2021). Additionally, taking into account the data obtained from PA questionnaires, we found that whereas PA levels were associated with both hemodynamic and structural parameters, PA indices were not independently associated with arterial stiffness (Gómez-García et al., 2021). In this regard, it would be valuable to identify to what extent accelerometer-derived indices are associated with arterial properties, independently of the exposure to other CRFs. Indices showing an independent association would be useful to indicate the expected values of arterial properties, regardless of other participants' characteristics [e.g., body mass index (BMI)].

In this context, working with healthy participants, we set the following aims: (i) to identify whether levels of accelerometer-derived PA indices depend on the recording site (hip vs. wrist) and/or the epoch length; (ii) to identify whether the levels of association between PA indices and arterial properties depend on the recording site or the epoch length; (iii) to assess to what extent the association between arterial properties and PA indices depend on the parameter (hemodynamic vs. structural vs. functional) or territory evaluated (central vs. peripheral, elastic vs. transitional vs. muscular arteries); (iv) to evaluate whether associations between PA indices and arterial properties are independent of other participants' characteristics. We analyzed both (i) the data obtained directly from the participants, and (ii) the data representing the deviation (z-score) of each arterial parameter from the expected value considering the age and sex of the participant (Castro et al., 2019, 2021; Gómez-García et al., 2021). Thus, our analysis focused not only on the levels of association between PA indices and arterial properties but also on the extent to which they would explain the level of deviation of the arterial system with respect to the values considered “ideal or optimal.”

## Methods

### Study Population

This study was carried out in the context of the Centro Universitario de Investigación, Innovación y Diagnóstico Arterial (CUiiDARTE) project (Bia et al., 2011; Santana et al., 2012a,b; Zócalo et al., 2020; Bia and Zócalo, 2021; Zócalo and Bia, 2021a,b, 2022). In this study, we included two samples. On the one hand, we worked with 60 volunteers who agreed to participate in a study protocol that included (i) recording of PA using accelerometers, (ii) clinical interview, (iii) anthropometric measurements, and (iv) cardiovascular evaluation. The volunteers were the first 60 who agreed to participate in the protocol among people who underwent cardiovascular studies at the CUiiDARTE Center as part of the project “Evaluación integral de la condición física y patrones de conducta sedentaria, actividad física y sueño, mediante ergoespirometría, bioimpedancia segmental multifrecuencia y acelerometría triaxial: asociación con el estado cardiovascular.” Only adults without chronic and infectious diseases who were not pregnant and who did not present atheromatosis (assessed by carotid and femoral ultrasound) were invited to participate. On the other hand, the study included a group of 1,688 healthy participants (“Reference group,” RG; male: 824, female: 864) without exposure to drugs or CRFs who were submitted to the same evaluation protocol used for the 60 volunteers (with the only exception of accelerometers recordings). This sample of participants was obtained from the CUiiDARTE Project Database (which includes participants from the general population, *n* = 3,619) and was allowed to determine, for each cardiovascular variable, the expected value in healthy participants (Zócalo et al., 2020; Bia and Zócalo, 2021; Zócalo and Bia, 2021a,b, 2022). That value represents the “optimal or ideal” based on age and sex. Data from the RG was used to obtain the equations that allowed to typify the cardiovascular parameters of the 60 participants studied with accelerometry (z-scores). Procedures were conducted in agreement with the Declaration of Helsinki and the protocol was approved by the Institution's Ethics Committee (Centro Hospitalario Pereira-Rossell, Hospital de Clínicas and Instituto Superior de Educación Física, Universidad de la República). Written informed consent was obtained prior to the evaluations and the use of data in research. The following describes the study protocol used in all participants and the accelerometer records obtained in the sub-sample of 60 volunteers.

### Anthropometric and Clinical Evaluation

A clinical interview, together with an anthropometric evaluation, enabled us to assess CRF exposure, defined according to the criteria (cut-off points) described later. A family history of CVD was defined by the presence of at least one first-degree relative with early CVD [<55 years (y) in men; <65 years in women]. Body weight (BW; Omron HBF-514C, Omron Healthcare, Inc., Illinois, USA) and body height (BH; portable stadiometer) were measured with the participants wearing light clothing and no shoes. BMI was calculated as a BW-to-squared BH ratio.

### Cardiovascular Evaluation

Participants were asked to avoid exercise, tobacco, alcohol, caffeine, and food intake 4 h prior to evaluation. Measurements were performed in a temperature-controlled environment (21–23°C), with the participant in the supine position and resting for at least 10–15 min. The cardiovascular evaluation included assessing hemodynamic, structural, and functional parameters. In this study, we focused on BP, beat-to-beat arterial diameter and wall thickness, and arterial stiffness parameters.

#### Peripheral and Central Blood Pressure

Heart rate, peripheral (brachial) systolic BP (baSBP), and diastolic BP (baDBP) were recorded (HEM-433INT; Omron Healthcare Inc., Lake Forest, IL, USA) immediately before, simultaneously, and/or after each non-invasive ultrasonographic and tonometric arterial measurement. Pulse pressure (baPP; baPP = baSBP – baDBP) and mean BP (baMBP, baMBP = baDBP + baPP/3) were calculated.

**Figure 1:**
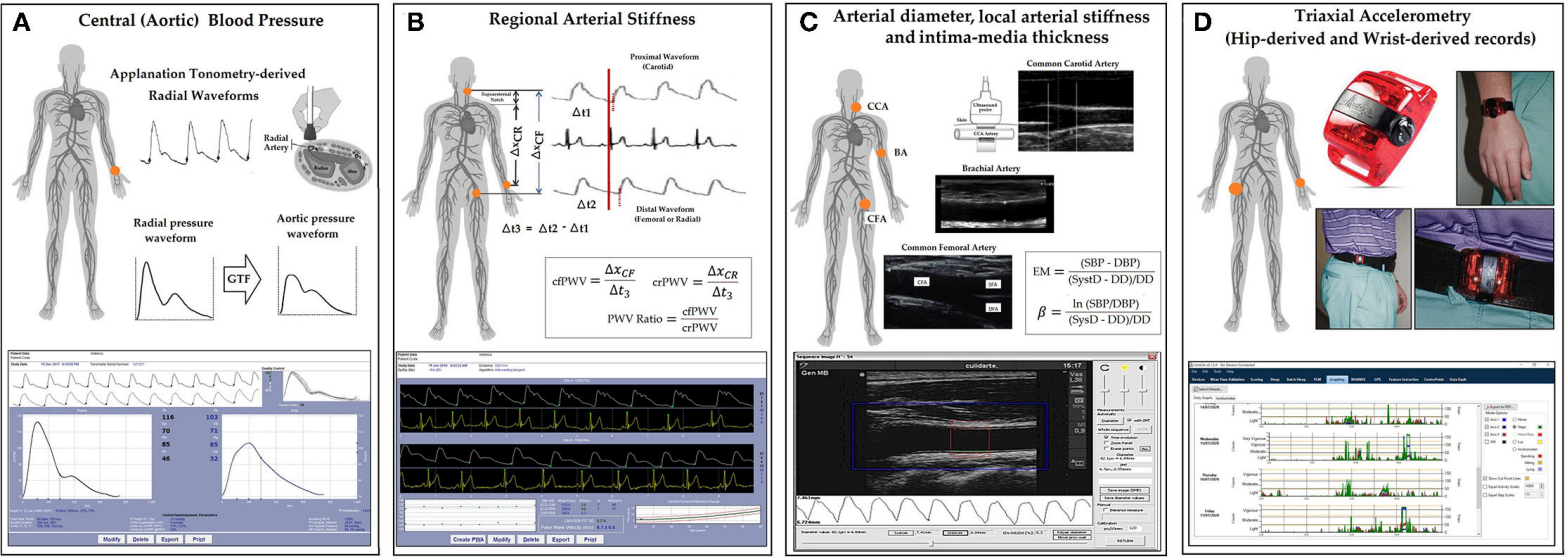
Summary of the non-invasive cardiovascular and accelerometry recordings approach performed on each participant. **(A)** Central aortic pressure measurements using SphygmoCor device and software (Applanation tonometry). GTF, general transfer function. **(B)** Regional arterial stiffness measurements using SphygmoCor device and software (Applanation tonometry). ΔxCR and ΔxCF: carotid-radial and carotid-femoral distances. Δt1 and Δt2: time between R-wave (QRS) and carotid and femoral “foot wave,” respectively. crPWV, cfPWV: carotid-radial and carotid-femoral pulse wave velocity. PWV, pulse wave velocity. **(C)** Arterial diameter, local stiffness, and intima-media thickness (IMT) measurements using echography and/or blood pressure measurement using echography and HemoDyn4M software. CCA, CFA, and BA, common carotid artery, common femoral artery, and brachial artery, respectively; EM, elastic modulus; β, beta or stiffness index; SBP and DBP, systolic and diastolic blood pressure; SysD and DD, peak systolic and end-diastolic arterial diameter; **(D)** Diagram of the placement site of the hip and wrist accelerometers (ActiGraph devices, model GT3X+, and ActiLife software).

Central (aortic) systolic and diastolic BP (aoSBP, aoDBP) levels were non-invasively obtained by applanation tonometry (SphygmoCor-CvMS, v.9, AtCor-Medical, Australia) (Zinoveev et al., 2019; Zócalo and Bia, 2022). Briefly, radial BP waveforms were obtained by tonometry and calibrated to baDBP and baMBP levels. Then, aoBP waveforms were derived from the calibrated waves using a radial-to-aortic general transfer function (Figure 1A).

#### Regional Arterial Stiffness and Central-to-Peripheral Arterial Stiffness Gradient

Carotid-radial (crPWV, a marker of upper-arm arteries stiffness) and carotid-femoral pulse wave velocity (cfPWV, a marker of aortic stiffness) were obtained by tonometry (Bia and Zócalo, 2021) (Figure 1B). PWV values that were shown correspond to the median of three records. The stiffness gradient (PWV ratio) was quantified using cfPWV/crPWV (Bia and Zócalo, 2021) (Figure 1B).

#### Arterial Diameter, Wall Thickness, and Local Arterial Stiffness

Left (L-) and right (R-) common carotid (CCA), common femoral (CFA), and left brachial (BA) arteries were analyzed using ultrasound (6–13 MHz, M-Turbo, Sonosite Inc., WA, USA). Sequences of images (30 s, B-Mode, longitudinal views) were stored for offline analysis. Diameter waveforms were obtained using border detection software (HemoDyn4M, Dinap s.r.l., Bs.As., Argentina). Peak systolic diameter (SysD), end-diastolic diameter (DD), and intima-media thickness (IMT; far wall, end diastole) values were obtained by averaging at least 20 beats. CCA diameter and IMT were measured a centimeter proximal to the bulb, CFA diameter was measured in a straight segment in the penultimate centimeter proximal to the bifurcation. BA measurements were acquired at the elbow level in a straight segment of at least one centimeter long (Marin et al., 2020) (Figure 1C).

Local stiffness was quantified by the elastic modulus (EM) and Beta Index (β). EM relates BP and diameter changes: EM = (SBP – DBP)/[(SysD – DD)/DD]. To minimize the impact of BP level on arterial stiffness, β was quantified: β = Ln(SBP/DBP)/[(SysD – DD)/DD]. Brachial BP was used to quantify CFA and BA EM and β, while aortic BP was used to quantify CCA EM and β (Figure 1C).

### Accelerometer Measurements and Data Reduction

Physical activity (PA) raw data was collected by ActiGraph accelerometers (GT3X+, ActiGraph, Pensacola, FL, USA), which measure accelerations in three axes (Y: vertical, X: horizontal, and Z: perpendicular) at a frequency of 100 Hz, with a dynamic range of ±8 units of gravity (ActiGraph, 2019). Participants were asked to wear two accelerometers. One was attached to an adjustable elastic belt with snap buckles and worn in line with the right hip. The other was placed in the non-dominant wrist (Trost et al., 2005) (Figure 1D). Participants were required to continue with their usual activities and to wear both accelerometers 24 h/day for at least 7 consecutive days, only taking them off for water activities (e.g., swimming, showering). Raw data were continuously stored in the device.

Anthropomorphic data (BH and BW), body location (hip or wrist), sex, race, and age of the participant were entered into the ActiLife software (v.6.13.4; ActiGraph, Pensacola, FL, USA) (Mueller et al., 2020). Data from the accelerometers was then downloaded and processed. As previously recommended (Migueles et al., 2017), we used the “normal” (default) filter, which was used in the validation study for the cut-off points or algorithms employed in our study (refer below) (Sasaki et al., 2011). In fact, we decided not to use the “Low Frequency Extension” option provided by ActiGraph, since it has been shown to have a large impact on the accelerometer outputs levels and to modify the records obtained at hip and wrist differently, which could have introduced an additional source of data variation (Tudor-Locke et al., 2015). For this study, raw acceleration data was converted into X, Y, Z axis, and vector magnitude (VM) activity counts at epoch lengths of 1, 5, 10, 30, and 60 s. The VM combines information recorded in 3-axes, rather than just the Y-axis [VM = √(X^2^+Y^2^+Z^2^)]. To be included in the data analysis, the participant needed to have at least 6 valid days, defined as those with ≥ 18 h of valid monitoring.

The intervals of use were identified by applying a wear-time validation algorithm described by Choi et al. (2011), which essentially selects flag periods of non-wear and filters them out from the analysis. We selected the default values for the optional criteria [e.g., “Small Window Length” equal to 30 min; “Spike Tolerance” (or motion artifact interval) equal to 2 min, use of VM rather than just the Y-axis] (Choi et al., 2011). Once the wear time data was validated, the following information was derived to score the PA: (i) PA-related Energy Expenditure (EE), (ii) Metabolic Equivalents, (iii) PA Bouts, (iv) PA Levels (“Cut Points”) and MVPA, (v) Steps, and (vi) Sedentary Analysis (Bouts and Breaks). Although a large number of PA-related indices were quantified (details of calculations and algorithms can be seen in Supplementary Material 1) and presented in the descriptive tables (data available for future comparisons), we focused on the four following indices: (i) EE (kcals/day), (ii) daily time in MVPA (%) (referred to as MVPA%), (iii) average steps/min, and (iv) VM counts-per-minute (CPM). Due to differences in wear time between days and participants, these indices were analyzed and expressed in percentages and/or the average of total daily wear time.

Physical activity (PA)-related EE was determined by the “Freedson VM3 Combination (2011)” algorithm, which combines (i) “Freedson VM3 ('11) formula” with (ii) “Williams Work-Energy ('98) equation” (when VM ≤ 2,453 CPM). “Freedson VM3 ('11)” equation uses all three axes to estimate EE. VM calculation is only valid if the epoch CPM exceeds the Scale × 2,453 CPM. Consequently, if VM > 2,453 CPM, then EE (kcals/min) = 0.001064^*^VM + 0.087512^*^BW – 5.500229 where BW is expressed in kg. If this is not achieved (VM ≤ 2,453 CPM), then the “Williams Work-Energy ('98)” formula is applied. The “Williams Work-Energy ('98)” formula utilizes the physics equivalent of energy: EE (kcals) = CPM^*^0.0000191^*^BW, where kcals are “total calories for a single epoch.” In turn, PA intensity was classified into 4 categories based on VM CPM, according to the “Freedson Adult VM3 (2011)” algorithm (Sasaki et al., 2011). Subsequently, we determined the MVPA% [i.e., the amount of time spent above the “Moderate PA” intensity cut-point level (2,690 CPM)], which indicates “significant” PA (Physical Activity Guidelines for Americans, 2008). ActiGraph uses a proprietary algorithm to count steps; process specifications are not available (Tudor-Locke et al., 2015).

The same PA indices (EE, MVPA%, steps/minute, and VM CPM) were calculated for hip and wrist records. Similar to previous studies, according to the manufacturer's recommendations, for wrist-worm data, the “Worn on wrist” option (ActiLife software) was selected during data scoring (McMinn et al., 2013; Mueller et al., 2020; Nuss et al., 2020; Guediri et al., 2021). When that option is selected, ActiLife software applies “piece-wise scaling” to the collected data, enabling direct comparison between PA indices (e.g., EE) obtained from hip and wrist measurements (ActiGraph, 2021). To this end, the “Worn on wrist” option converts counts obtained at the wrist [“wrist counts,” (WC)] into “hip equivalent counts” (HEC), using manufacturer's calibration equations (published on the manufacturer's website): (i) WC range: 0–644, HEC = 0.5341614^*^WC; (ii) WC range: 645–1,272, HEC = 1.7133758^*^WC – 759.414013; (iii) WC range: 1,273–3,806, HEC = 0.3997632^*^WC + 911.501184; and (iv) WC range: 3,807 to infinity, HEC = 0.0128995^*^WC + 2383.904505 (Mueller et al., 2020; ActiGraph, 2021). The resultant counts (HEC) are then used to calculate EE and MVPA% using equations and cut-off points validated for data obtained at the hip or waist [e.g., Freedson VM3 ('11) formula used to calculate EE was developed from data obtained with waist-worn devices] (Sasaki et al., 2011). The conversion equations, generally used by those using ActiGraph wrist-mounted devices, come from the manufacturer's internal research and developments (ActiGraph, 2021). It is to be noted that, in theory, when the “Worn on wrist” option is selected, EE and MVPA% values obtained from wrist and hip recordings (for a participant in a given period) should be similar. However, this is a controversial issue that is still discussed and under investigation (Mueller et al., 2020; Nuss et al., 2020; Guediri et al., 2021). It is also to note that the “Worn on wrist” option does not modify the step/minute and VM CM data obtained at the wrist (Mueller et al., 2020).

We selected these four accelerometer-derived indices because they are among the most studied and recommended in cardiovascular health studies. For instance, both the American Heart Association (American Heart Association, 2019) and the American College of Sports Medicine (Garber et al., 2011) developed PA guidelines with recommendations related to volumes and intensities of EE. The global “age-specific” PA recommendations of the WHO are based on MVPA% (World Health Organization, 2020). On the other hand, for several years now, different organizations (e.g., U.S. President's Challenge Physical Activity and Fitness Awards Program) have based their recommendations on “step count” (Tudor-Locke et al., 2011). Finally, the VM CPM was analyzed to obtain a continuous variable directly related to the accelerometer recordings, without being determined by modeling or by the participant characteristics. Thus, the PA indices considered included both indices whose calculations depend on the use of the “Worn on wrist” option (EE and MVPA%) and indices that do not depend on the selection of that option (steps/minute and VM CPM) (McMinn et al., 2013; Mueller et al., 2020; Nuss et al., 2020; Guediri et al., 2021).

### Statistical Analysis

**Figure 2:**
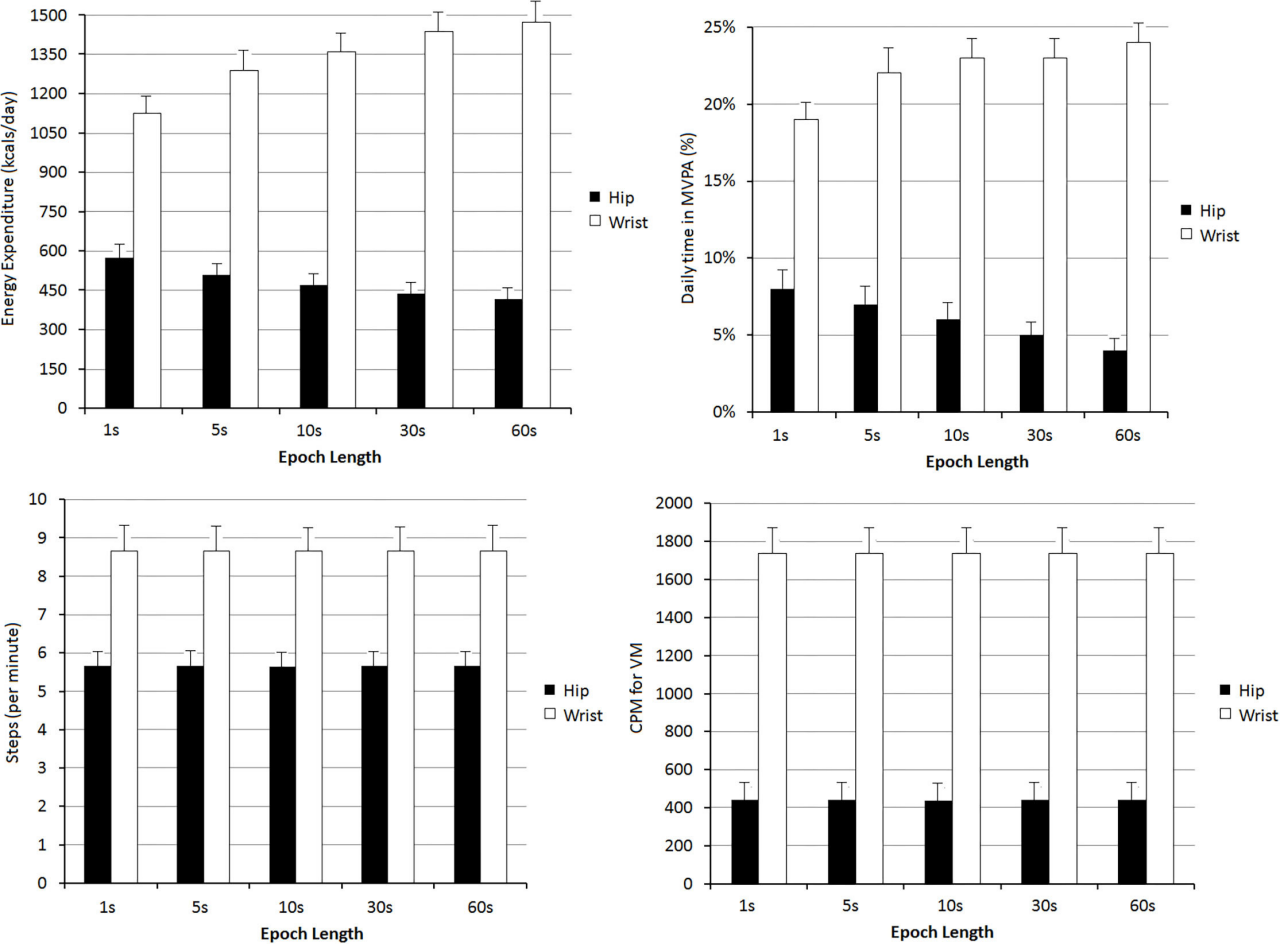
Levels of energy expenditure, daily time in moderate-to-vigorous physical activity (MVPA%), steps per minute and vector magnitude (VM) counts per minute (CPM), found depending on the registration site (hip or wrist) and the epoch length: 1, 5, 10, 30, and 60 s. All comparisons between hip and wrist records were statistically significant (*p* < 0.05). For the same recording site (hip or wrist), the levels of Energy Expenditure and daily time in MVPA% were different (*p* < 0.05) when comparing the values obtained with different epoch lengths.

A stepwise analysis was performed. First, descriptive statistics were obtained for the 60 participants with recordings with accelerometry (Table 1; Figure 2; Supplementary Tables 1–10 in Supplementary Material 2).

**Table 1:** Characteristics of participants (*n* = 60).

**Variables**	**MV**	**SD**	**Min**.	**P25th**	**p50th**	**p75th**	**Max**.
**Demographic, anthropometric, and cardiovascular risk factors**							
Age (years)	36.72	10.03	23	29	34	43	62
Sex (Female, %)	42.90						
Body height (m)	171.31	9.14	150	164	169	178	189
Body weight (Kg)	76	15	55	66	74	81	115
BMI (Kg/m^2)^	25.84	3.44	20.6	23.55	24.61	28.58	33.97
Current smoker (%)	5.70						
Hypertension (%)	2.90						
Dyslipidemia (%)	8.60						
Obesity (%)	9.00						
Family history of CVD (%)	8.60						
Anti-hypertensive (%)	2.90						
Total cholesterol (mg/dl)	222	37	187	187	217	262	262
HDL cholesterol (mg/dl)	66	12	58	58	59	80	80
LDL cholesterol (mg/dl)	132	48	84	84	132	180	180
Tryglicerides (mg/dl)	121.67	9.02	113	113	121	131	131
Glicaemia (mg/dl)	88	3	86	87	88	91	92
Creatinine (mg/dl)	0.75	0.07	0.7	0.7	0.75	0.8	0.8
**Central (aortic) and peripheral (brachial) blood pressure**							
aoSBP (mmHg)	106.46	8.96	92	99	107	114	125
z-aoSBP (SD)	−0.08	0.86	−1.78	−0.73	−0.33	0.66	1.41
aoDBP (mmHg)	73.74	7.64	58	68	73	80	85
z-aoDBP (SD)	0.16	0.89	−1.92	−0.46	0.1	0.92	1.63
baSBP (mmHg)	122.21	10.76	100	113	121	130	144
z-baSBP (SD)	0.17	0.96	−2.32	−0.61	0.03	0.86	2.14
baDBP (mmHg)	73.32	7.69	56	70	73.5	78	90
z-baDBP (SD)	0.25	0.94	−2.18	−0.37	0.33	0.79	2.22
Heart rate (beats/minute)	63.74	9.29	47	57	66	69	86
**Arterial structural (diameters, wall thickness) parameters**							
Left CCA DD (mm)	6.51	0.62	5.47	6.14	6.43	7.11	7.55
z-Left CCA DD (SD)	0.11	1.06	−1.68	−0.43	0.01	1.01	1.75
Left CCA IMT (mm)	0.59	0.12	0.42	0.51	0.58	0.66	0.96
z-Left CCA IMT (SD)	0.08	1.19	−1.7	−0.78	−0.22	0.99	3.49
Right CCA DD (mm)	6.64	0.57	5.37	6.27	6.65	6.92	8.51
z-Right CCA DD (SD)	0.15	0.96	−2.08	−0.47	0.22	0.62	3.21
Right CCA IMT (mm)	0.59	0.11	0.41	0.52	0.56	0.64	0.93
z-Right CCA IMT (SD)	0.09	1.13	−1.65	−0.51	−0.24	0.75	2.73
Left CFA DD (mm)	7.75	1.46	5.74	6.74	7.33	8.72	11.86
z-Left CCA DD (SD)	−0.13	1.16	−1.72	−0.86	−0.43	0.7	2.93
Right CFA DD (mm)	7.63	1.56	5.19	6.53	7.33	8.6	12.79
z-Right CFA DD (SD)	−0.27	1.3	−2.4	−1.07	−0.57	0.43	3.95
Left BA DD (mm)	3.78	0.59	2.9	3.19	3.82	4.29	4.8
z-Left BA DD (SD)	−0.07	0.83	−1.42	−0.77	−0.04	0.73	1.44
**Local arterial stiffness (Elastic Modulus and Beta Index)**							
Left CCA EM (mmHg)	696.98	173.51	444.38	538.76	695.79	822.11	1086.19
z-Left CCA EM (SD)	−0.22	0.81	−1.49	−0.88	−0.39	0.27	1.84
Left CCA Beta Index	7.15	1.47	4.16	5.98	7	8.48	9.84
z-Left CCA Beta Index (SD)	−0.31	0.65	−1.64	−0.84	−0.33	0.05	0.99
Right CCA EM (mmHg)	702.96	195.3	432.66	585.65	660.1	808.68	1175
z-Right CCA EM (SD)	−0.1	0.91	−1.38	−0.71	−0.09	0.24	2.37
Right CCA Beta Index	7.22	1.77	4.52	5.86	7.1	7.93	11.55
z-Right CCA Beta Index (SD)	−0.2	0.76	−1.26	−0.82	−0.07	0.25	1.65
Left CFA EM (mmHg)	1096.3	428.23	433.02	693.58	1079.19	1377.39	2134.8
z-Left CFA EM (SD)	−0.24	0.71	−1.28	−0.9	−0.24	0.22	1.43
Left CFA Beta Index	11.37	4.19	4.72	7.91	11.16	14.19	22.36
z-Left CFA Beta Index (SD)	−0.31	0.66	−1.31	−0.89	−0.35	0.14	1.44
Right CFA EM (mmHg)	1024.88	397.76	376.27	713.3	1047.14	1185.78	2337.89
z-Right CFA EM (SD)	−0.27	0.81	−1.49	−0.97	−0.16	0.01	2.42
Right CFA Beta Index	10.53	3.7	4.23	7.96	10.56	11.77	23.1
z-Right CFA Beta Index (SD)	−0.33	0.73	−1.41	−0.81	−0.36	−0.07	2.15
Left BA EM (mmHg)	1503.8	1036.75	345.58	702	1067.29	2280	3759.43
z-Left BA EM (SD)	0.29	1.49	−1.35	−0.8	−0.33	1.44	3.61
Left BA Beta Index	15.42	10.24	3.75	8.19	10.35	21.31	40.01
z-Left BA Beta Index (SD)	0.2	1.41	−1.41	−0.78	−0.5	1.03	3.66
**Regional arterial stiffness (Pulse Wave Velocity)**							
cfPWVcf “real” (m/s)	7.63	1.41	5.72	6.43	7.52	8.13	12.27
z-cfPWV “real” (SD)	0.13	1.04	−2.04	−0.72	0.16	0.93	2.39
crPWV (m/s)	10.38	1.46	7.7	8.9	10.3	11.75	13
z-crPWV (SD)	0.41	1.2	−1.75	−0.41	0.36	1.58	2.62
**Arterial stiffness central-peripheral gradient (cfPWV/crPWV)**							
PWV Ratio	0.74	0.16	0.48	0.62	0.7	0.8	1.13
z-PWV Ratio (SD)	−0.22	1.3	−2.69	−1.2	−0.3	0.44	2.68

*MV, mean value; SD, standard deviation; P25th, p50th, p75th, percentile 25, 50, and 75, respectively; Min., minimal value; Max, maximal value; BMI, body mass index; CVD, cardiovascular disease; Z-, z-score; aoSBP, aoDBP, aortic systolic and diastolic blood pressure; CCA, CFA, BA, common carotid, common femoral, and brachial artery, respectively; DD, arterial end-diastolic diameter; IMT, intima-media thickness; EM, elastic modulus; cfPWV and crPWV, carotid-femoral and carotid-radial pulse wave velocity; PWV, pulse wave velocity*.

#### Agreement Between Accelerometry-Derived Data: Body Site and Epoch Length Analysis

**Figure 3:**
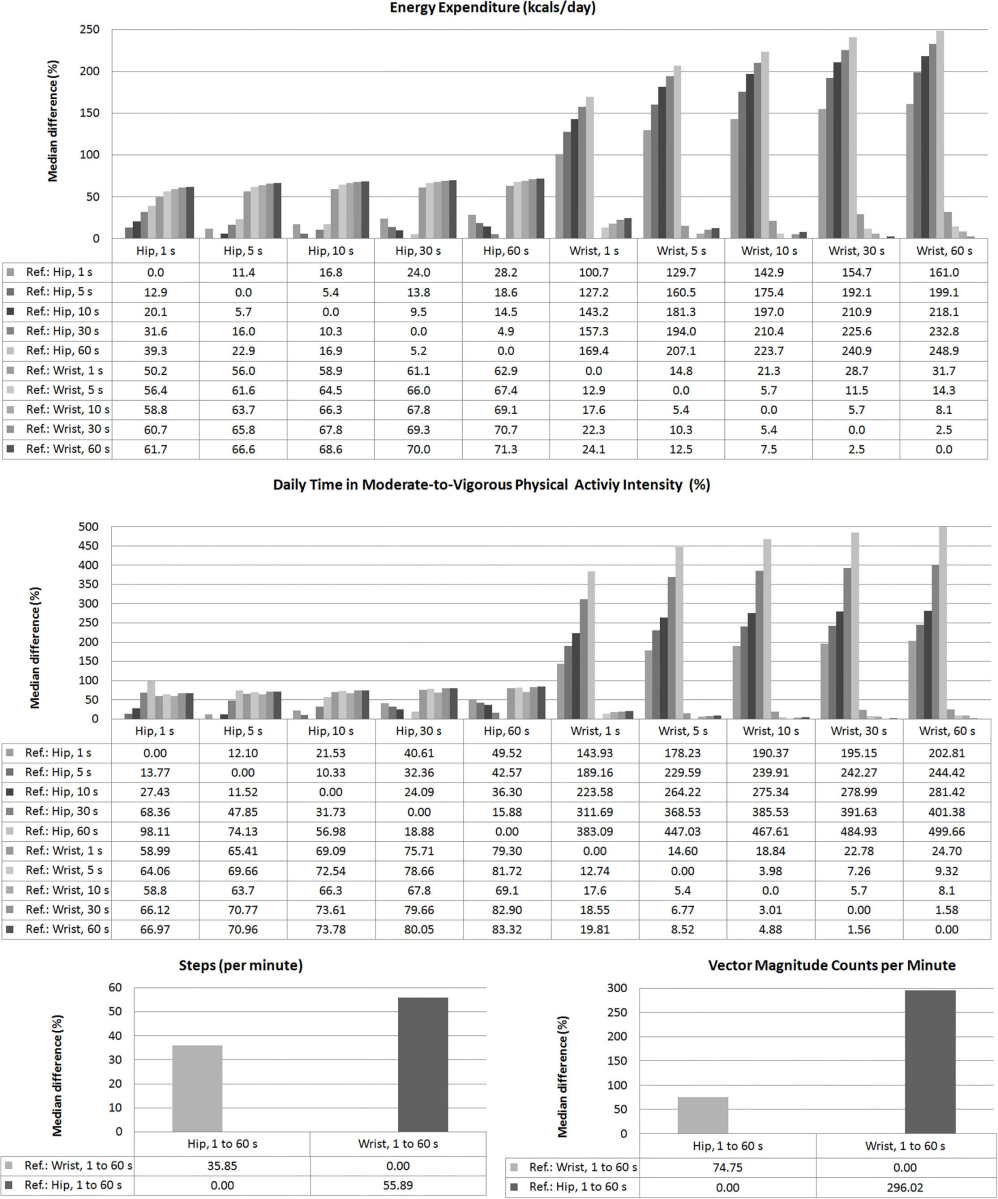
Relative differences (%), median levels, between Energy Expenditure, daily time in Moderate-to-Vigorous Physical Activity (MVPA, %), steps/min, and VM CPM found depending on the registration site (hip or wrist) and the epoch length: 1, 5, 10, 30, and 60 s. Levels of statistical significance (*p*-values) for each paired comparison are detailed in Supplementary Tables 15–18 in Supplementary Material 2.

Second, we analyzed the degree of agreement between PA indices obtained from different recording sites and considered different epoch lengths. To this end, correlation analysis (Supplementary Tables 11–14 in Supplementary Material 2) and Bland–Altman tests (Figure 3; Supplementary Tables 15–18 in Supplementary Material 2) were considered. Although no significant association level was found in 22 of the 324 correlations evaluated, the Bland-Altman tests were performed for all paired comparisons. As in previous studies, Bland-Altman analysis was done using “Krouwer's method” (Krouwer, 2008; Ruiz et al., 2011; Chastin et al., 2018). This method allows, for a given value obtained with a particular method (“reference method”), to know the difference with respect to another method (“alternative method”). The difference between the methods is expressed on the y-axis and the “reference value” on the x-axis [instead of the mean, as in the most widely used (classical) Bland-Altman analysis]. When the described variant is considered, a linear regression equation defining the relationship between the “reference method” (x value; e.g., EE, hip record, epoch: 1 s) and the difference with respect to the “alternative method” (e.g., EE, wrist record, epoch: 1 s) is obtained. The analysis can be applied (as in our article) with the aim of identifying whether there is a proportional error between the methods and determining the expected value for “alternative method” as a function of data obtained with the method considered “reference.” The method provides a predictive equation, which allows knowing from a certain recording site and epoch length what would be obtained using another recording site and/or epoch length. Considering the above, in our analyses, the different recording sites (wrist and hip) and epoch lengths were considered as “reference” (and in turn, as “alternative”). This allowed two different simultaneous comparisons: between recording sites (hip vs. wrist), and among epochs lengths (1 vs. 5 vs. 10 vs. 30 vs. 60 s). In each Bland-Altman test, the existence of systematic (mean) and proportional (that varied depending on the “reference” value) errors were evaluated. To this end, the corresponding linear regression equations were obtained (Supplementary Tables 15–18 in Supplementary Material 2).

#### Standardized Cardiovascular Variables (z-Scores)

Third, cardiovascular variables were expressed as z-scores. As was mentioned, participants to be included in the RG were selected from the CUiiDARTE database (Supplementary Table 19 in Supplementary Material 2). None of the participants included in the RG meet any of the following (exclusion criteria) (Zócalo and Bia, 2021a,b, 2022): history of CVD; use of BP, lipid, or glucose-lowering drugs; arterial hypertension; current smoking; diabetes; dyslipidemia; and/or obesity. None of the participants in the RG had congenital, chronic, or infectious diseases or cardiac arrhythmias.

Once the RG was built, age-related equations for mean value (MV) and SD were obtained. We implemented parametric regression methods based on several models (fractional polynomials, polynomial, and ratios of polynomials) (Zócalo et al., 2020; Bia and Zócalo, 2021; Zócalo and Bia, 2021a,b, 2022). This procedure provides age-related equations for each model. The one with the best-fit was chosen to calculate the z-scores (Table 1) for each cardiovascular variable from participants who had accelerometer-derived records (*n* = 60) (Supplementary Table 20 in Supplementary Material 2). A z-score is a dimensionless number obtained by subtracting the observed value from the RG MV and dividing the result by the RG SD. The z-score describes the position of a raw score in terms of its distance from the MV (“expected or optimal” value) when measured in SD units.

#### Accelerometry-Derived Indexes: Association With Arterial Characteristics

**Figure 4:**
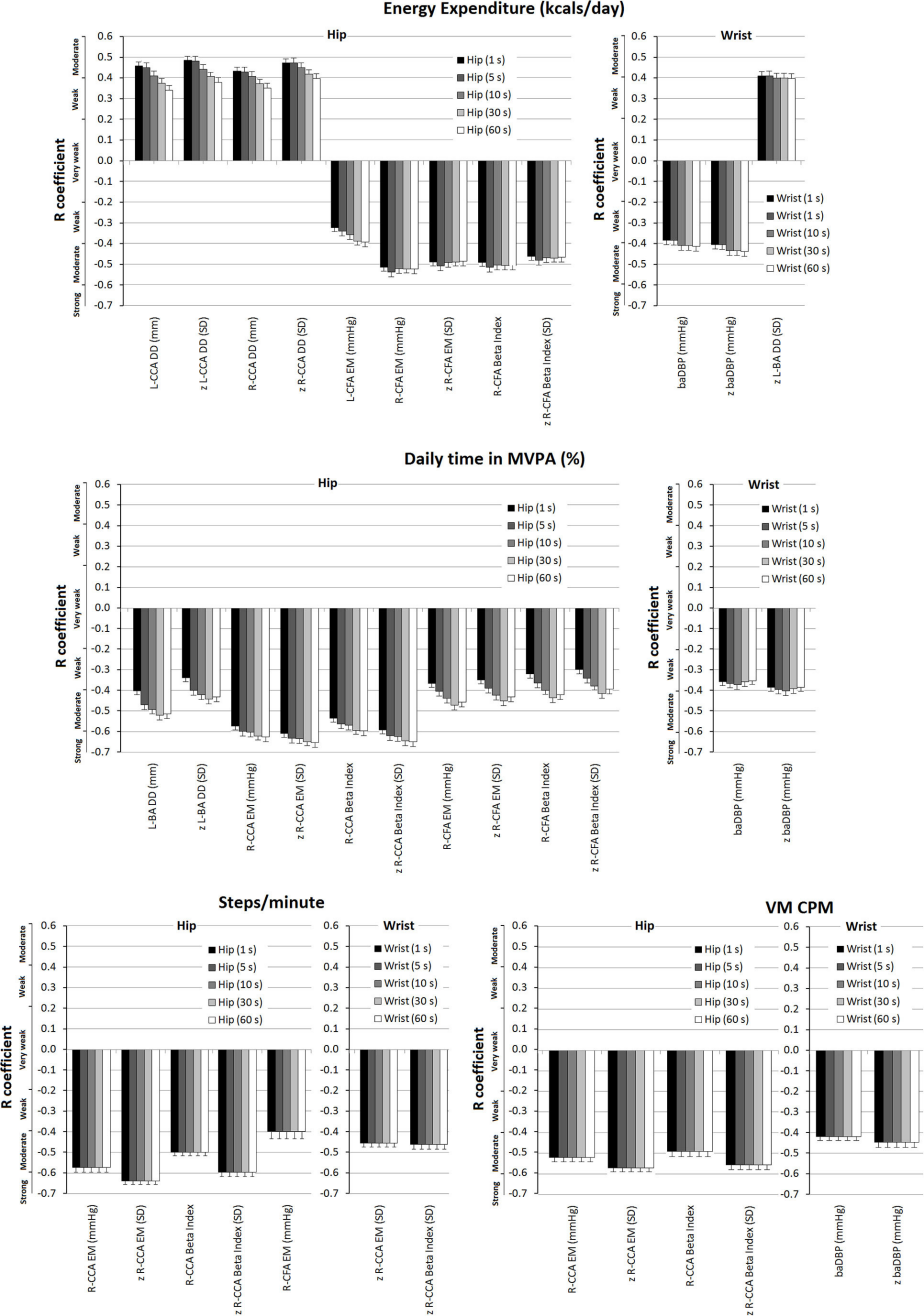
Strength of association (*r* coefficients; partial correlations) between accelerometry-derived physical activity indices and cardiovascular properties (measured levels and/or z-scores), for hip and/or wrist records, as a function of epoch length (1, 5, 10, 30, and 60 s). Only the associations in which statistical significance was reached for at least one epoch are plotted. A more extensive description can be found in Supplementary Tables 25–28 in Supplementary Material 2.

Simple bivariate and partial correlations (adjusting for confounders including sex, age, BMI, hypertension, current smoke, dyslipidemia, and family history of CVD) analyses were done to quantify the association between accelerometry-derived indices and cardiovascular parameters, expressed both as un-standardized and standardized (z-scores) variables (Figure 4; Supplementary Tables 21–28 in Supplementary Material 2). Evans's Empirical Classification (“correlation strength”) was used for *r* interpretation: <0.20, very weak; 0.20–0.39, weak; 0.40–0.59, moderate; 0.60–0.79, strong; ≥0.80, very strong (Evans, 1996).

#### Sample Size and Statistical Package

According to the central limit theorem, a normal distribution was considered (considering Kurtosis and Skewness coefficients and number of studied participants, with sample size >30) (Lumley et al., 2002). Considering an α = 0.05 (type I error), β = 0.20 (type II error), and *r* = 0.35–0.50 (effect size for correlation analysis), the number of participants included was higher than the minimum sample size required, both to construct the RG (sample size required: 377), and to perform agreement and association analyses (sample size required: 29–60). Analyses were done using SPSS Software (v.26, IBM-SPSS Inc., Chicago, IL, USA), MedCalc (v.14.8.1, MedCalc Inc., Ostend, Belgium), and NCSS 2020 (NCSS, Kaysville, UT; www.NCSS.com). A *p* < 0.05 was considered statistically significant.

## Results

### Participants' Characteristics

Table 1 shows the characteristics of the participants who had accelerometers-derived measurements. Note the balanced sex distribution and low exposure to CRFs. There were no participants with diabetes, use of anti-hyperlipidemic, or anti-diabetic agents. In addition, considering the exclusion and inclusion criteria none of the participants had CVD. Cardiovascular variables showed the expected distributions and trends. For example, aoSBP levels were lower than baSBP levels and CCA arteries stiffness was lower than that of peripheral arteries (CFA and BA) (Table 1).

### Agreement Between Accelerometry-Derived Data: Recording Site and Epoch Length

Data from hip records were lower than those obtained with wrist-based accelerometers (Figure 2; Supplementary Tables 1–10 in Supplementary Material 2).

Steps/min and VM CPM data obtained with hip and wrist devices did not vary with the epoch length considered. However, as the epoch length increased, EE and MVPA% levels obtained with hip-accelerometers decreased. The opposite was observed when analyzing wrist-accelerometer data (Figure 2; Supplementary Tables 1–10 in Supplementary Material 2).

For any of the PA indices, the differences observed when comparing recording sites were greater than the obtained when considering different epoch lengths (Figure 3). In fact, regardless of the epoch length analyzed, the differences between data obtained from a given recording site considering different lengths (e.g., 39% between hip EE data when comparing epoch lengths of 1 and 60 s) were never as high as those observed when comparing data from different recording sites. A more extensive quantitative analysis can be found in Supplementary Tables 11–18 in Supplementary Material 2.

Bland-Altman tests showed statistical significance for systematic errors, both when considering data from different locations or when comparing epoch lengths. In addition, with some exceptions, there were also proportional errors both when comparing epoch lengths or different recording sites. Consequently, besides the differences between the recording sites and epoch lengths, the differences varied depending on the levels of the variables measured (Supplementary Tables 15–18 in Supplementary Material 2).

### Accelerometry-Derived Indexes and Arterial Properties: Size, Direction, and Nature (Dependent vs. Independent) of the Association

#### Central and Peripheral Blood Pressure

Partial correlations showed “weak to moderate” negative associations between wrist-derived EE data and baDBP (*r*: −0.384 to −0.413) and z-baDBP (*r*: −0.405 to −0.439) (Figure 4). Similarly, a negative association (“weak to moderate”) between MVPA% (wrist recordings) and baDBP (*r*: −0.353 to −0.373) was observed only after adjustments were made (Figure 4; Supplementary Table 26 in Supplementary Material 2). In turn, after adjustment, VM CPM (wrist-recordings) was negatively associated with baDBP (*r*: −0.419) and z-baDBP (*r*: −0.446) (Figure 4; Supplementary Table 28 in Supplementary Material 2).

#### Arterial Structural Parameters

Energy expenditure (EE; hip; and wrist data) showed positive associations (mostly “moderate to strong”) with CCA and CFA diameters. In addition, EE from wrist-recordings was positively associated with BA DD (Supplementary Table 21 in Supplementary Material 2). After adjustment, the association (“moderate”) between EE (hip-recordings) and Left CCA DD (*r*: 0.341–0.458), z-Left CCA DD (*r*: 0.377–0.486), Right CCA DD (*r*: 0.451–0.434), and z-Right CCA DD (*r*: 0.398–0.472) remained significant (Figure 4; Supplementary Table 25 in Supplementary Material 2). On the other hand, after adjustment, the positive association between EE (wrist-data) and diameters was no longer significant, with the only exception of z-left BA DD (Figure 4).

Daily time in moderate-to-vigorous physical activity (MVPA%) analysis (hip-recordings) supports the existence of positive independent relationships between PA and central arteries diameters since both before and after adjustment there were trends (*p* between 0.05 and 0.10) for positive associations with Left CCA DD and z-CCA DD (Supplementary Tables 22, 26 in Supplementary Material 2). MVPA% from wrist-recordings did not show significant association (or a trend to) with CCA, CFA, or BA structural parameters (Supplementary Tables 22, 26 in Supplementary Material 2).

Neither before nor after adjustment for cofactors the relationship between steps/min and structural parameters reached statistical significance. A similar finding was observed when considering VM CPM obtained from hip and wrist recordings (Supplementary Tables 23, 24, 27, 28 in Supplementary Material 2).

#### Local Arterial Stiffness

After adjusting for confounders, EE data from wrist-recordings was no longer associated with local stiffness, whereas EE levels (hip-recordings) showed “moderate” negative associations with Right CCA and Right and Left CFA stiffness (Figure 4; Supplementary Table 25 in Supplementary Material 2). After adjustment, MVPA% (wrist-recordings) showed no association with local stiffness. On the contrary, MVPA% data obtained from hip-recordings remained negatively associated with CFA and CCA (right hemibody) stiffness (“moderate to strong” association) and kept the trend to be negatively associated with BA stiffness (Figure 4; Supplementary Table 26 in Supplementary Material 2). Some associations were no longer significant after adjusting for cofactors; mainly the negative associations of steps/min and VM CPM (hip-records) with CCA stiffness were those that remained significant (“moderate to strong”) (Figure 4; Supplementary Tables 27, 28 in Supplementary Material 2).

#### Regional Arterial Stiffness and Stiffness Gradient

Regional stiffness and PWV ratio showed no independent associations with EE or MVPA% (hip and wrist recordings). Neither before nor after the adjustment for cofactors of the VM CPM data from hip or wrist-recordings were associated with regional stiffness or stiffness gradient (PWV Ratio).

Regardless of the recording site, before adjusting for confounders, there was no association between steps/min and cfPWV, crPWV, or PWV ratio. However, after adjustments, the steps/min data obtained from hip-recordings showed a trend (*p* between 0.05 and 0.10) toward negative associations with z-cfPWV, PWV Ratio, and z-PWV Ratio (Supplementary Table 27 in Supplementary Material 2).

### Accelerometer-Derived Indices and Arterial Characteristics: Comparison of Central vs. Peripheral, and Structural vs. Functional vs. Hemodyamic Cardiovascular Parameters

**Figure 5:**
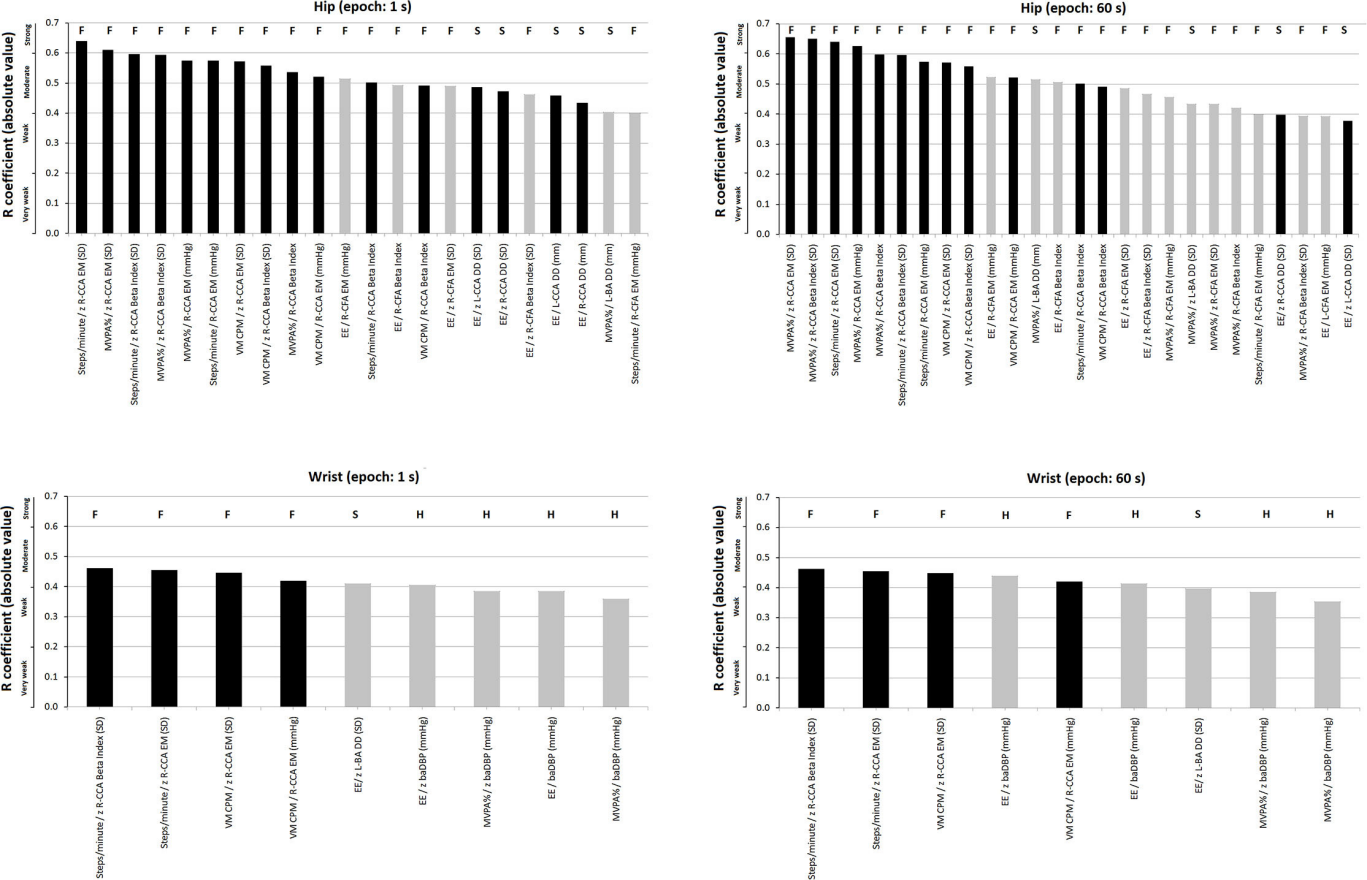
Strength of association (*r* coefficients absolute value; partial correlations), ranked from highest to lowest “r,” between accelerometry-derived physical activity indices and cardiovascular properties (measured levels and/or z-scores). Separate charts are presented for wrist and hip, and 1- and 60-s epoch. Only the associations in which statistical significance was reached are plotted. Black and gray bars: central (e.g., common carotid artery, CCA) and peripheral [e.g., common femoral artery (CFA) or brachial artery (BA)] parameters, respectively. The letters above each bar indicate whether it corresponds to a functional (F), structural (S), or hemodynamic (H) parameter. A more extensive description can be found in Supplementary Tables 25–28 in Supplementary Material 2.

Disregarding the recording site, the arterial variables with the strongest levels of independent association with PA indices were those of the central arteries (i.e., CCA DD and stiffness) (Figure 5, Supplementary Tables 25–28 in Supplementary Material 2). Regardless of the recording site or epoch length considered, the highest levels of independent association were observed for functional (e.g., CCA stiffness) rather than hemodynamic or structural parameters (Figure 5, Supplementary Tables 25–28 in Supplementary Material 2).

There were accelerometer-derived PA indices associated with arterial parameters of more than one arterial territory. EE and MVPA% recorded at the hip were associated with both CFA and CCA local stiffness. On the contrary, other indices only showed association with functional properties of a single arterial territory (e.g., VM CPM recorded at hip or wrist only showed association with CCA local stiffness parameters) (Figure 5; Supplementary Tables 25–28 in Supplementary Material 2).

### Association Between Accelerometer-Derived PA Indices and Arterial Characteristics: Influence of the Recording Site (Hip vs. Wrist)

Some PA indices and arterial characteristics were independently associated with disregard of the accelerometer location (e.g., VM CPM and CCA stiffness), whereas other associations depended on the recording site considered (e.g., EE or MVPA% and baDBP were only associated when wrist-data were considered). Compared to indices derived from wrist recordings, those obtained from hip measurements showed a higher number of statistically significant independent associations with arterial parameters (hip vs. wrist: 28 vs. 9 associations, respectively) (Supplementary Tables 25–28 in Supplementary Material 2).

Regardless of the PA index considered, there were always several statistically significant independent associations when hip recordings and central artery parameters were considered. In contrast, wrist recordings only showed associations with CCA when considering steps/min and VM CPM.

### Association Between Accelerometry-Derived Indices and Arterial Characteristics: Influence of the Epoch Length

In some cases, the variation in the epoch length resulted in(significant) changes in the levels of association between an accelerometry-derived PA index and an arterial parameter. For instance, in partial correlations, the associations between EE data obtained from hip-recording and Left or Right CCA DD decreased (from “moderate” to “weak”) as the epoch length increased (Figure 4) (Supplementary Table 25 in Supplementary Material 2). In turn, the opposite was observed when analyzing the association between EE (wrist-recording) and baDBP, or the relationship between MVPA% and local (CCA or CFA) stiffness (Supplementary Tables 25, 26 in Supplementary Material 2). In fact, the associations between MVPA% (hip-recording) and Right CCA EM increased (from “moderate” to “strong”) as the epoch length increased (Figure 4; Supplementary Table 25 in Supplementary Material 2).

In contrast, when considering steps/min and VM CPM, the association strengths were shown to be unchanged as epoch length was varied (Figure 4; Supplementary Table 25 in Supplementary Material 2).

## Discussion

### Agreement Between Accelerometry-Derived Data: Recording Site and Epoch Length

Our findings are in agreement with previous studies in which using the same devices (ActiGraph GT3X+) and processing option provided by the software (“Worn-to-wrist”), it was observed that compared to data derived from wrist-recordings, those obtained from hip records showed lower (i) PA-related EE (Guediri et al., 2021), (ii) MVPA% (Kamada et al., 2016), (iii) steps counts (Hildebrand et al., 2014; Tudor-Locke et al., 2015; Kamada et al., 2016; Migueles et al., 2017; Mandigout et al., 2019), and (iv) VM counts (Kamada et al., 2016). Additionally, our data showed that the differences would depend on the epoch length considered. As the epoch length increased, EE and MVPA% levels decreased when considering hip recordings, while the opposite was observed for wrist-derived data. In turn, steps/min and VM CPM values did not vary in association with variations in the epoch length considered (Figure 2). Consequently, for EE and MVPA% levels, the differences (discrepancies) between data from hip and wrist increased when selecting longer epoch lengths. In fact, for a given recording site, variations in the epoch length considered resulted in mean systematic and proportional errors (Figure 3).

The epoch length-related differences observed in this study are consistent with previous data obtained in children and adolescents using the same accelerometers (hip or waist recordings) (Aadland et al., 2018, 2020; Altenburg et al., 2021). In this regard, the reduction in MVPA% observed in hip-derived data as the epoch length increased agrees with Aadland et al. (2018, 2020) who reported that in children, the daily time in MVPA (minute/day) was reduced from 76 ± 23 to 74 ± 25 and 65 ± 28 when considering 1, 10, and 60-s epoch lengths, respectively. Additionally, the above is also consistent with the findings of Altenburg et al. (2021). These authors analyzed the impact of 5, 15, and 60-s epoch lengths on PA and sedentary behavior data in healthy children (*n* = 902, 5–12 years). The authors found that light PA, bouts of MVPA, and sedentary behavior increased with longer epochs. Besides, total MVPA decreased using longer epochs (total time: 60, 55, and 44 min/day for 5, 15, and 60-s epochs, respectively) which is in agreement with the findings of this study [total time in MVPA (MV): 67.9, 46.7, and 38.98 min/day for 5, 30, and 60-s epoch length, respectively] (Supplementary Tables 1–10 in Supplementary Material 2). This has further strengthened the hypothesis that epoch length could modify the strength of association between accelerometer-derived indices and cardiovascular variables. Consequently, as stated, differences in data acquisition and processing could lead to significant differences in findings (and conclusions) when addressing PA and cardiovascular system association.

Despite different acceleration patterns expected to be obtained from wrist and hip recordings, some accelerometers (e.g., Actigraph GT3X) are offered, highlighting that the device can be used to evaluate the same parameters (e.g., MVPA%) regardless of the accelerometer location (ActiGraph, 2019). The latter must be entered into the software to define the “filters and algorithm” to be used to estimate the PA indices and to make data obtained from different recording sites comparable and/or well-matched. For instance, to make wrist records compatible with hip records, mathematical options like ActiLife wrist correction (“Worn on wrist” option) are available. If this is omitted (e.g., due to an error in processing data and/or unawareness of the issue), the information recorded at the wrist would be erroneously processed as if it had been recorded at the hip or waist (by default), achieving (generally speaking) higher levels of EE and MVPA%. As expected, this device-dependent processing scheme makes the calculations more complex (and less clear), since, first, the software uses non-linear and activity-dependent “wrist-hip” calibration schemes (“wrist-to-hip transformation”) to artificially “reconcile” the acceleration patterns obtained at different locations to finally use scoring methods that were developed using hip accelerometers data in most commercially available devices. To date, no study has evaluated the validity of software data-correction despite its significance in terms of data analysis, validity, and comparability. In addition, in general, the algorithms are not available but are properties of the manufacturer (Mandigout et al., 2019). In this context, it should be noted that since the level of PA is performed regardless of how it is measured, it is clear that in a given individual and condition, the mathematical algorithms should provide the same results for accelerometer-derived indices or outcome of interest (e.g., EE, time in MVPA, steps/min) regardless of the device-location (e.g., allowing the individuals to assess their PA and compliance with the international recommendations) (Guediri et al., 2021).

Given that we used the “Worn to wrist” option (approach recommended by the manufacturer) and found participants to be “more active” based on wrist data, it could be said that wrist correction would not make data from different locations alike. Consequently, it could be proposed that, despite using the “Worn to wrist” option, hip and wrist-derived PA and epoch length-derived indices (with the exception of steps/min and VM CPM) would not provide similar values and would not be equivalent (Figures 2, 3). Hip and wrist recordings should not be used interchangeably, and the epoch length should be selected in advance according to the PA index of interest. Our findings agree with and complement the results of previous works and emphasize that caution is needed when using EE, MVPA%, steps count, and VM CPM values recorded at wrist and hip as outcomes to assess or characterize PA, since different information is obtained. It has been suggested that users may assume that the “Worn on wrist” option in ActiLife would provide more accurate PA estimates (Mueller et al., 2020). However, as was analyzed, our results and those of other authors warn about the accuracy of the estimates (McMinn et al., 2013; Mandigout et al., 2019; Mueller et al., 2020; Nuss et al., 2020). In this context, it is noteworthy that the use of wrist correction factors will likely decrease as new wrist-based algorithms are developed.

In line with the above, when evaluating the association between PA indices and arterial parameters, it was necessary to separately analyze PA data obtained from different locations and epoch lengths. To our knowledge, there are no previous studies analyzing the associations of different arterial parameters, types, and regions with accelerometry-derived parameters.

### Accelerometry-Derived Indices and Arterial Properties: Size, Direction, and Nature (Dependent vs. Independent) of the Association

#### Central and Peripheral Blood Pressure

As was mentioned, statistically significant negative associations between wrist-derived EE data and baDBP and z-baDBP were only observed after adjusting for cofactors. Then, regardless of age, sex, and exposure to CRFs, higher EE (obtained from wrist recordings) was associated with lower baDBP (absolute values, in mmHg) and lower baDBP with respect to the expected (RG value) for age-matched healthy participants not exposed to CRFs. As was described, EE calculus (kcals/min) considers the relationship between the VM (or CPM) and BW (Kg) of a participant. At least in theory, when adjusting (partial correlations) for participant's characteristics (e.g., BW as BMI determinant), VM (or CPM) would become the variable that governs the relationship between EE and baDBP, making it evident that the higher the PA detected in the wrist, the lower the baDBP (potential beneficial effect of PA on BP associated to reduced peripheral vascular resistances and structural arterial changes). In contrast, when no adjustments are made (simple bivariate correlations), the participant's anthropomorphic characteristics would “hide and reverse” the association, making EE positively associated with aoSBP (e.g., related with the known positive association between aoSBP and BMI) (Garcia-Espinosa et al., 2018) and no longer associated with baDBP. Similarly, a negative association (“weak to moderate”) between MVPA% (wrist recordings) and baDBP was observed only after adjustments were made. Thus, regardless of other characteristics of the participant, higher MVPA% (wrist-recordings) was associated with (beneficial) variations in baDBP (values even lower than those expected in the RG). Although it did not reach statistical significance, after the adjustment for cofactors (but not before), the steps/min level showed a tendency (*p* between 0.05 and 0.10) to be negatively associated with baDBP (wrist and hip recordings) and z-baDBP (wrist recordings). In turn, VM CPM data from wrist-recordings were negatively and independently associated with baDBP and z-baDBP. The above, obtained for steps/min and VM CPM, indices that do not require considering “cut-off values” and/or equations that include variables related to individual characteristics (as is required to calculate MVPA% or EE), reinforces that in healthy participants from the general population, higher “movement” is associated with lower baDBP (largely determined by peripheral vascular resistances; lower resistances, lower baDBP).

The described association suggests that when assessing the association between PA and BP levels, the use of an accelerometry-derived index that, in its calculation, takes into account anthropometric characteristics (e.g., EE) could have the disadvantage of integrating into a single index—variables that have inverse relationships with BP. In this regard, the use of indices whose determinants do not include BW or BMI (e.g., VM CPM) could be more useful for identifying the beneficial negative relationship between PA and BP, minimizing the impact of confounding factors.

#### Arterial Structural Parameters and Local Arterial Stiffness

The PA-related EE determined from hip-recordings would be positively and independently associated, mainly with central (carotid) arteries diameters (Figure 4). Since the associations remained significant after adjustments, they would not be mediated solely by the recognized positive association between arterial diameters and BMI, as might be the case for CFAs (Sandgren et al., 1999; Garcia-Espinosa et al., 2018). Moreover, the MVPA% analysis (hip-records) supports the existence of positive independent relationships between PA and central arteries diameters since there were trends (*p* between 0.05 and 0.10) in both before and after adjustment for positive relationships with Left CCA DD and z-CCA DD. In contrast, in general terms, after adjustment, the positive association between EE (wrist-records) and arterial diameters was no longer significant. In turn, MVPA% data from wrist measurements did not show significant associations (or trends to) with carotid, femoral, or brachial structural parameters. Therefore, indices from wrist recordings would not be independently associated with characteristics of arteries that are the main determinants of arterial impedance and left ventricular load.

These results show that the structural characteristics of central arteries (i.e., CCA) would be the most sensitive to structural variations associated with variations in the accelerometry-derived indices EE and MVPA%. Therefore, the analysis of central (e.g., CCA) rather than peripheral (e.g., CFA and BA) arteries would be the most valuable for monitoring structural arterial variations associated with PA levels (determined by accelerometry-derived indices).

After adjusting for confounders, EE (wrist-records) was no longer associated with local stiffness, whereas EE levels obtained from hip-recordings showed “moderate” negative associations with CCA and CFA local stiffness (Figure 4). In line with the above are findings obtained when analyzing MVPA%. After adjusting for cofactors, MVPA% levels obtained from wrist-recording showed no association with local arterial stiffness. This again suggests that accelerometry-derived indices obtained from wrist-recordings would not be independently associated with the main biomechanical arterial characteristics. On the contrary, after adjustment for confounders, MVPA% data obtained from hip-recordings remained negatively associated with CFA and CCA (right hemibody) stiffness (reaching “moderate to strong” levels of association) and kept the trend to be negatively associated with BA stiffness. Consequently, whatever the participants' characteristics, higher levels of MVPA% (hip-recordings) were associated with lower local arterial stiffness. The described above for EE and MVPA% is similar to the obtained when analyzing steps/min and VM CPM. Although some relationships were no longer significant after adjusting for cofactors, the negative associations between data from hip-recordings and CCA stiffness were, mainly, those that remained significant.

Then higher PA levels obtained from hip-recordings (but not from wrist-measurements) would be (or tend to) independently associated with lower levels of femoral and carotid stiffness (which would certainly indicate a PA-related benefit for the cardiovascular system). The above reinforces the value of hip-recordings to assess the association between PA and arterial characteristics.

Each arterial segment fulfills two different but interrelated “biomechanical” functions: to deliver an adequate blood supply to organs and tissues (“conduit function”), and to smooth the pressure and flow pulsation resulting from the intermittent left ventricular ejection so as to transform the oscillatory (highly pulsatile) hemodynamic into a continuous low-pulsatile one (“buffering or cushioning function”). Both functions are mainly determined by the cross-sectional area of the arterial segment and by the arterial wall stiffness. Arteries with a larger diameter and lower stiffness interpose less resistance to blood flow (more efficient “conduction function,” which is to say, lower local or characteristic impedance) while performing greater filtering (a more efficient “buffering function”) of blood flow and pressure pulsatility. In this context, our results showed that accelerometer-derived indices assessed at the hip (but not at the wrist; even though the “Worn on wrist” option was used) would be related to the levels of arterial diameter and stiffness, which would mean that they would be independently associated with the two main determinants of the arterial biomechanical functions.

#### Regional Arterial Stiffness and Central-Peripheral Stiffness Gradient

Regional stiffness and PWV ratio showed no independent associations with EE, MVPA%, or VM CPM data obtained from hip and wrist recordings. However, after the adjustment for cofactors, steps/min data obtained from hip-recordings showed a trend toward negative associations with z-cfPWV, PWV Ratio, and z-PWV Ratio. Accordingly, higher PA levels measured in terms of steps/min would tend to be associated with lower regional aortic (central) stiffness and “central stiffness/peripheral stiffness” ratio (would gradually move toward values below 1). Reduced aortic stiffness levels would be beneficial to the cardiovascular system as they result in lower levels of ventricular afterload (cardiac effects) and increased buffering capacity. In turn, a reduced PWV ratio would protect the microcirculation and contribute to ensuring an adequate capillary-tissue exchange, reducing the amount of pulsed energy accessing the exchange system (Bia and Zócalo, 2021; Pereira et al., 2021).

In a large cohort sample [*n* = 2,455, 47 ± 9 years; (53% women)], Andersson et al. (2015) found that higher MVPA% levels were negatively associated with cfPWV and forward pressure wave and positively associated with left ventricular mass. Differences in the PA levels recorded in this study and that in Andersson et al.'s study could contribute to explaining the (relative) dissimilar findings. About this, whereas in this study the mean value of daily MVPA was 46.72 min, the authors reported a mean value equal to 29.9 and 25.5 min for men and women, respectively. In a cross-sectional study in British men, Parsons et al. (2016) found that accelerometer-derived PA indices were associated with arterial stiffness, augmentation index, and CCA IMT. Unfortunately, sample characteristics or methodological differences between studies preclude accurate comparisons.

Finally, as an overall message, from the above four sub-sections, it could be stated that: first, when the associations were evaluated adjusting for co-factors, the number of significant associations was reduced. Then, many associations between PA indices and cardiovascular properties would be mediated or influenced by variations in other factors (mediation/moderation). Whereas, after adjusting for cofactors, BP no longer showed a positive association with some PA indices but became negatively associated (wrist data) with EE and MVPA% (which would be related to the proposed benefits of PA on BP). CCA diameters and stiffness continued to show, respectively, positive and negative associations with PA, reinforcing that the relationship between these variables would not depend on participant's characteristics or exposure to other CRFs (Figure 4). Accordingly, it could be said that independent of confounding factors, accelerometry-derived PA indices would be globally associated with an arterial system showing reduced impedance to blood flow (lower stiffness and larger arterial diameter), which in turn could contribute to the trend toward lower BP levels.

### Accelerometer-Derived Indices and Arterial Characteristics: Comparison of Central vs. Peripheral and Structural vs. Functional Cardiovascular Parameters

Since accelerometer-derived PA indices showed a major independent association with central elastic (i.e., CCA) than with peripheral arteries (i.e., CFA and BA), the former would be preferentially “affected” or would be more “sensitive” to the PA effects (Figure 5; Supplementary Tables 25–28 in Supplementary Material 2). This observation could have important practical implications. First, the assessment of PA impact on the arterial system should focus on central arteries. Identifying that accelerometry-derived PA indices are mostly associated with central artery property levels open the possibility of using them to monitor interventions that are expected to impact on true left ventricular afterload (Zócalo et al., 2013). In turn, peripheral arteries (e.g., BA) could give inaccurate data regarding the impact of PA on the vascular system (Dinenno et al., 2001). In this context, it is noteworthy that exercise training demonstrated beneficial effects on the femoral artery. In this regard, although the impact of exercise on the established peripheral arterial disease would be different, the observed association between PA activity and CFA diameters could contribute to explaining the benefits of PA in the context of vascular adaptations to training and/or disease (Dinenno et al., 2001; Haas et al., 2012). In this regard, our data do not show an independent association between PA indices and CFA DD, but rather a relationship that would depend on other factors (only observed in simple correlations). Prospective interventional studies would be necessary to confirm the above.

On the other hand, after adjustment for cofactors, regardless of the recording site or epoch length considered, the highest levels of association were observed for functional (e.g., CCA local stiffness) rather than hemodynamic or structural arterial parameters (Figure 5, black columns; Supplementary Tables 25–28 in Supplementary Material 2). The above is in agreement with Baumgartner et al. (2020) who described that in children and adolescents, accelerometer-derived PA indices had differences in their associations with functional and structural arterial properties, and consequently, not all accelerometer-derived PA indices would have the same ability to identify inter-individual cardiovascular variations.

Regardless of epoch length, there were accelerometer-derived PA indices associated with the arterial parameters of more than one arterial territory. On the contrary, other indices only showed association with functional properties of a single territory. This could have a practical impact on the selection of accelerometry indices to be used in studies (cross-sectional or longitudinal) that attempt to identify associations between PA levels and the cardiovascular system. The selection of indices associated with more than one characteristic and/or arterial pathway could increase the depth and scope of the study approach, whereas selecting indices without an association could lead to wrong conclusions (e.g., that PA is not associated with “arterial characteristics”). The above reinforces the idea that PA does not impact homogeneously the arterial system, but rather differentially affects the arterial territories and characteristics. Regarding the latter, local stiffness indices (e.g., EM) would be more sensitive than regional parameters (cfPWV or crPWV) in showing associations with accelerometry-derived indices. Consequently, when analyzing and discussing the impact of PA on “arterial stiffness” or “arterial diameter,” it is necessary to specify the territory and parameter evaluated.

### Association Between Accelerometer-Derived PA Indices and Arterial Characteristics: Influence of the Recording Site (Hip vs. Wrist)

Our results indicated that compared to wrist-derived indices, those obtained from hip measurements showed a greater number of statistically significant associations with arterial parameters. In theory, this could be evidence that the “acceleration” recorded at the hip would be more closely related to PA levels that are biologically linked to structural and functional characteristics of the arterial system. As an example, when walking, running, bending down, or climbing stairs, the aerobic PA associated with benefits on the cardiovascular system could be more accurately recorded with an accelerometer placed on the hip. As discussed, although the software used “counts” measured at the wrist and after converting them to hip equivalents (conversion equations) and calculated PA parameters that attempt to be similar to those from hip-recordings, these clearly did not outperform the data obtained directly from hips in terms of levels of association with arterial characteristics. Anyway, whether the observed differences between hip and wrist data (in terms of association with arterial properties) are such as to recommend the use of hip recordings over wrist should be further analyzed in prospective studies.

Unfortunately, there are no previous studies comparatively analyzing the associations between accelerometer-derived indices (hip and wrist) and arterial variables. We only found one manuscript. In this regard, our findings agree with Cooke et al. who described that the association between step counts and cfPWV was only observed when the accelerometer was attached to the hip (−0.28 m/s, 95% CI −0.58, 0.01) (Cooke et al., 2018).

### Association Between Accelerometry-Derived PA Indices and Arterial Characteristics: Influence of the Epoch Length

Our results indicate that although the length of the epoch could determine the levels of association between PA indices and arterial characteristics, it is not possible to make generalizations regarding the appropriateness of using a particular epoch length to increase the overall levels of association between accelerometer-derived PA indices and arterial characteristics. Defining the “best” epoch length is beyond the scope of this work. As mentioned, the results showed that the levels of association between accelerometry-derived indices and cardiovascular variables can both increase and decrease with increasing epoch length, which could depend on the recording site and index considered. This heterogeneity in the impact of epoch length on the strength of association between accelerometry-derived indices and arterial variables underscores the complexity of the issue. In this context, it could be said that the elective methodological approach, parameters to be analyzed, and associations to be assessed would vary and should be individually defined depending on the aim of the study. Further studies should address to what extent systematization or consensus on epoch lengths (defined considering the above) could improve the usefulness of accelerometry-derived variables in the analysis of the relationship between PA and the status of the cardiovascular system.

In practical terms, the selected “epoch length” could determine the association between PA levels and cardiovascular health markers. As was mentioned, Aadland et al. (2018, 2020) found that in children the associations between accelerometer-derived PA levels and metabolic health markers (i.e., a composite metabolic health score) differed among different epoch lengths. About this, the associations between moderate-intensity PA (2,000–4,000 counts/min) and metabolic health markers observed for data analyzed using 60-s epoch were significantly weakened when shorter epoch durations were used (e.g., when considering 1-s epochs, that is, a configuration with sufficient resolution to capture accurately vigorous PA) (Aadland et al., 2020). The authors' findings suggested the associations between moderate-intensity PA and metabolic health could be spuriously high when analyzing data using long epochs. This could be explained by the misclassification of vigorous PA as moderate when averaging PA over longer periods (Aadland et al., 2018). In addition, when considering the 60- vs. 1-s epoch setting, the PA intensity associated with metabolic health was found to be significantly left-skewed; the strongest associations with metabolic health were observed for 7,000–8,000 cpm (rather than for 4,000–5,000 cpm) (Aadland et al., 2020). Then, conscious use of epoch settings would be critical for accurate analysis and understanding of the relationship between PA and health status (Aadland et al., 2018, 2020). The findings of the authors both question and challenge researchers' knowledge on how PA is distributed and accumulated and on how accelerometer data should be managed and analyzed (Aadland et al., 2020). In line with what was described for metabolic health indicators, our findings reinforce the significance of the epoch length considered for adequate analysis and interpretation of data on the association between accelerometer-derived PA indices and the structural, functional, and hemodynamic properties of central and peripheral arteries.

### Strengths and Limitations

This study has strengths and limitations that should be considered. First, our study included a comprehensive non-invasive evaluation of cardiovascular properties. Second, the definition of an RG made it possible to determine inter-individual cardiovascular variations (z-scores). Since the RG included Uruguayan adults non-exposed to CRFs, at the time of identifying in the 60 participants the degree of deviation from the expected or optimal values, we avoided using bibliographic data obtained non-Uruguayan participants who do not necessarily have characteristics similar to those of the Uruguayan population. Third, in this study, central and peripheral SBP and DBP were used to quantify central and peripheral arterial stiffness levels.

We are aware that our research may have limitations. First, it is a cross-sectional study, so participants were not followed and the temporal profiles of the cardiovascular properties and/or the time spent on PA components and subcomponents were unknown. Second, the accelerometers do not allow capturing some PA (e.g., bicycling). Third, an obvious limitation was the lack of a criterion measure, such as direct observation, which precluded comparing and determining which epoch length would be the most accurate to assess PA. It should be noted that in addition to the mentioned (e.g., classification techniques based on accelerometer “intensity cut points”) and used in this study, there are other analysis and classification methodologies (e.g., machine learning techniques or algorithms) proposed to determine PA behaviors from tri-axial wrist and hip acceleration raw data which allow the development of models (i.e., classifiers) (Kerr et al., 2016, 2017). However, in the present study, we limited the analysis to the approaches described (i) to focus the scope and limit the extension of the study, (ii) because they are among the most used so far, and (iii) because they are available for data processing using Actigraph GT3X +/ActiLife software. This is not a minor issue since previous studies showed differences in PA behavior estimates across hip and wrist locations, which varied depending on the way data was processed. Similarly, it should be noted that different computational analysis methods had different strengths depending on the type of measure being analyzed (Kate et al., 2016). Fourth, we did not consider thigh location as a wear option. This was simply to facilitate the protocol, as the inclusion of a third recording site would have reduced the project's ability to succeed. The value and eventual superiority of other accelerometer locations in terms of their ability to provide information about PA and its association with cardiovascular status should be analyzed in future studies. Fifth, our results were obtained in healthy participants and may not necessarily correspond to findings that could be obtained in other populations. The approach was selected based on the fact that our motivation was to assess the relationship between PA and vascular properties in participants considered healthy, given that we aimed to identify and analyze the association between PA and the vascular system from a physiological and preventive perspective. Sixth, we did not record ambulatory heart rate in order to obtain a “real” indication of PA intensity. Finally, as in previous studies, Bland-Altman analysis was done using “Krouwer's method” (Krouwer, 2008; Ruiz et al., 2011; Chastin et al., 2018). We are aware of the fact that this approach is not the classic one and although it has strengths, it also has limitations. In this regard, it should be noted that the “reference” method is the one with a smaller measurement error compared to the other measure evaluated, and strictly, this is not the case for data in our study.

## Conclusions

Working with healthy adults in free-living conditions, our study contributes to the knowledge that differences in data acquisition and processing could lead to differences in results when addressing the association between accelerometer-derived PA indices and the cardiovascular system. First, despite using the “Worn to wrist” option provided by the manufacturers of ActiGraph devices, accelerometer-derived PA indices obtained from hip-recordings were lower than those from wrist-measurements. Additionally, these differences varied as a function of the accelerometry-derived PA levels recorded (proportional errors). Second, the EE and MVPA% levels obtained from wrist and hip change in the opposite way when varying the epoch length. As the epoch length increased, EE and MVPA% levels decreased when considering hip recordings, but the opposite was observed for wrist-derived data. Steps/min and VM CPM values did not vary in association with variations in the epoch length considered. Hip and wrist-derived PA indices and indices obtained considering different epoch lengths would not provide similar data and would not be considered equivalent. Then, hip and wrist data should not be used interchangeably. In turn, the epoch length should be selected in advance according to the proposed aims or analysis (e.g., the PA index of interest). Third, mostly central arteries (CCA) characteristics and mainly the functional ones (local stiffness) were associated with accelerometry-derived indices (EE, MVPA%, steps/min, and VM CPM). Then, central territories would be those mainly related to PA levels assessed by accelerometry. Whatever the index considered (EE, MVPA%, steps/minute, or VM CPM), there was an association with central arteries (CCA). Fourth, hip records (with respect to wrist records) showed the highest level of association with arterial characteristics. In general, PA indices obtained by hip recordings outperformed those obtained from wrist measurements in terms of association with arterial parameters. Fifth, variations in the epoch length considered resulted in variations in the strength of association between arterial characteristics and EE and MVPA% levels (but not steps/min and VM CPM), although there was no clear trend enabling to define whether a certain epoch length would be “of choice.”

## Data Availability Statement

The raw data supporting the conclusions of this article will be made available by the authors, without undue reservation.

## Ethics Statement

The studies involving human participants were reviewed and approved by Comité de Ética del Hospital de Clínicas, Comité de Ética del Centro Hospitalario Pereira-Rossell y Comité de Ética del Instituto Superior de Educación Física. Written informed consent to participate in this study was provided by the participants' legal guardian/next of kin.

## Author Contributions

MG-G, DB, and YZ contributed to the conception and design of the study, performed the accelerometry and cardiovascular non-invasive recordings, constructed and organized the database, and performed the statistical analysis. MG-G, JT, DB, and YZ wrote the first draft and final version of the manuscript, contributed to manuscript revision, read, and approved the submitted version. All authors contributed to the article and approved the submitted version.

## Funding

This research was funded by Agencia Nacional de Investigación e Innovación (ANII), grant number PRSCT–008–020 and FSPI_X_2015_1_108484, and extra budgetary funds provided by DB, YZ, and CUiiDARTE Center.

## Conflict of Interest

The authors declare that the research was conducted in the absence of any commercial or financial relationships that could be construed as a potential conflict of interest.

## Publisher's Note

All claims expressed in this article are solely those of the authors and do not necessarily represent those of their affiliated organizations, or those of the publisher, the editors and the reviewers. Any product that may be evaluated in this article, or claim that may be made by its manufacturer, is not guaranteed or endorsed by the publisher.

## Abbreviations

aoDBP: Aortic diastolic blood pressure
aoSBP: Aortic systolic blood pressure
BA: Brachial artery
baDBP: Brachial artery diastolic blood pressure
baSBP: Brachial artery systolic blood pressure
BH: Body height
BMI: Body mass index
BP: Blood pressure
BW: Body weight
CCA: Common carotid artery
CFA: Common femoral artery
cfPWV: Carotid-femoral pulse wave velocity
CPM: Counts per minute
CRFs: Cardiovascular risk factors
crPWV: Carotid-radial pulse wave velocity
CVD: Cardiovascular disease
DD: End-diastolic arterial diameter
EE: Energy Expenditure
EM: Elastic modulus
IMT: Intima-media thickness
MV: Mean value
MVPA: Moderate-to-vigorous physical activity
PA: Physical activity
PWV Ratio: Pulse wave velocity ratio (cfPWV/crPWV quotient)
RG: Reference group
SD: Standard deviation
SysD: Peak systolic arterial diameter
VM: Vector magnitude
z-: Z-score
β: Beta index.
