# Supplementary material for: Influence of Epoch Length and Recording Site on the Relationship Between Tri-Axial Accelerometry-Derived Physical Activity Levels and Structural, Functional, and Hemodynamic Properties of Central and Peripheral Arteries
Source: Front Sports Act Living. 2022 Feb 24;4:799659. doi: 10.3389/fspor.2022.799659 (PMC8909126; doi:10.3389/fspor.2022.799659)
Supplement: Supplementary file 2 [file Data_Sheet_1.docx]

Supplementary File 1

# Accelerometer Measurements, Data Collection and Processing

Physical activity (PA) raw data was collected by ActiGraph devices (GT3X+, ActiGraph accelerometers, Pensacola, Florida, USA), which measure accelerations in three different axes (vertical, horizontal, perpendicular) at a frequency of 100 Hz in each axis, with a dynamic range of ±6 units of gravity.

Participants were asked to wear two accelerometers, one of which was attached to an adjustable elastic belt with snap buckles and worn in line with the right hip according to evidence-based guidelines, while the second was placed on their non-dominant wrist (Trost et al., 2005). Both accelerometers were worn 24 hours/day (including while sleeping), except during water activities (e.g., bathing, swimming, showering). Raw data was stored on the device continuously (24 hours) for seven complete days, while subjects were performing their normal daily activities. As a data analysis inclusion citeria, each subject needed at least six valid days, which were defined as having ≥18 hours of valid monitoring (e.g., the device was being worn).

The wear intervals were identified with the ´Wear Time Validation tool´ by applying a wear time validation algorithm described by Choi et al. (Choi et al., 2011), which essentially selects flag periods of non-wear, and filters them out from the analysis. More precisely, the algorithm utilizes a forward and backward looking windowing technique to better categorize non-wear times, while evaluates the activity level obtained for the single Vector Magnitude (VM; a parameter that combines the information recorded in 3-axes, rather than just the Y-axis {VM=√[X2+Y2+Z2]}), where X, Y and Z are medial-lateral, vertical and antero-posterior axes. As previously recommended, we selected the default values for the optional criteria (e.g., a ´Small Window Length´ equal 30 minutes, a ´Spike Tolerance´ [or Artifactual movement interval] equal 2 minutes, with VM rather than just the Y-axis) (Choi et al., 2011).

Subsequently, the ActiGraph’s digital filter feature (band-limits frequency range: 0.25 to 2.5 Hz) within the Actigraph Analysis Tool of the software (version 6.10.2) was used for the following aims: 1) to detect normal human motion while rejecting accelerations changes outside the pass band; 2) to identify and remove non-human like motion. Then, the raw data (of wear time) is summed into chunks of data called "epochs" and the acceleration values were converted to “activity counts", which are then used for calculus and generate data figures. The value of the counts will vary based on: (i) the frequency, and (ii) the intensity of the raw acceleration. For this study, raw acceleration data was converted into vertical axis and VM activity counts at epoch lengths of 1-, 5-, 10-, 30-, and 60-seconds (s). A broad range of epoch lengths was established in order to perform a comprehensive methodological comparison and clarify their most appropriate use. Importantly, while progressive longer epoch times reflect the average activity level during that period resulting in progressive “smoothing effects” (Trost et al., 2005), total volume of activity accumulated per day would be not affected. Indeed, the epoch length only becomes an issue when PA characterization represents the outcome of interest (Edwardson et al., 2010).

Finally, once the recorded data were validated, the following information was derived to characterize (“scoring”) the PA: (i) Energy Expenditure (EE), (ii) Metabolic Equivalents (METs), (iii) PA Bouts (PA), (iv) PA Levels (´Cut Points´) and Moderate-to-Vigorous PA (MVPA), (v) Steps/minute, and (vi) Sedentary Analysis (Sedentary Bouts and Breaks).

# Physical Activity Assessment

## Energy Expenditure

Energy Expenditure (EE) was determined by the ´Freedson VM3 Combination (2011)´ algorithm, which combines the (i) ´Freedson VM3 ('11) formula´ with the (ii) ´Williams Work-Energy ('98) equation´ (when the VM is ≤2453 counts per minute [CPM]).

The ´Freedson VM3 ('11)´ equation uses all three axes to estimate EE. However, the VM calculation is only valid if the epoch CPM exceed the Scale×2453. Consequently, if VM >2453 CPM, EE (kcal/min) = 0.001064*VM+0.087512*BW-5.500229, where BW is body weight (kg). If this is not achieved (VM ≤2453 CPM), then the ´Williams Work-Energy ('98)´ formula is applied.

The ´Williams Work-Energy ('98)´ formula utilizes the physics equivalent of energy: EE (kcal) =CPM*0.0000191*BW, where kcal is ´total calories for a single epoch´. The linearity of this equation allows ActiLife Software to scale non-60 s epochs up to their 60s equivalent then back down again to obtain a per-epoch kcal value.

By using this approach, PA-related EE was expressed as follows: (i) net or total (considering the entire recorded period or data set), (ii) daily average (´average kcals/day´) and (iii) hour average (´average kcals/hour´).

## Metabolic Equivalents (METs).

METs are defined as the caloric consumption of an active subject compared with the resting basal metabolic rate (BMR), and are used to estimate the functional capacity. For instance, one MET (1 kcal/kg/hour) corresponds to the caloric consumption of a person while at complete rest (the BMR or ´normal sedentary energy´), while METs >1 METs represent activities associated with higher energy consumption, such as light walking, doing household chores (e.g., 2-4 METs), running (e.g., >4 METs), etc.

To quantity METs rate for a period of time, using CPM data, we use ´Freedson Adult (1998)´and ´Freedson Children (2005)´ equations, for subjets ≥19 and ≤18 y, respectively (Freedson et al., 1998, 2005). Using ´Freedson Adult (1998)´ equation kcals are calculated using the ´Freedson Combination EE algorithm´(see above). Once the total kcal are calculated for the epoch, they are scaled up to an hourly equivalent, and MET Rate calculated as = (hourly kcal equivalent)/BW (Freedson et al., 1998). Using ´Freedson Children (2005)´ equation, MET Rate is calculated as follows: 2.757+(0.0015*CPM)-(0.08957*Age)-(0.000038*CPM*Age; age in y (Freedson et al., 2005). Using this approach, MET Rate was quantified as the hour average (considering the entire recorded period). Of note, for the purpose of this study, since adults were only included, only adult-related equations were utilized.

## Physical Activity Bouts

A ´bout of PA or PA bout´ (to differentiate it from a sedentary behavior bout), was defined using the ´Freedson 1998 algorithm´, which considers (i) Minimum Counts (per minute) equal 1952 CPM (this sets the lower threshold required to detect the start of, or continuation of a bout); (ii) Maximal Counts (per minute) of 50,000 CPM (arbitrary definition since the PA performed by a subject can never exceed this value); (iii) a Minimum Time (Length) equals to 10 minutes; (iv) a Drop Time equal to 2 minutes; the Drop Time value acts as a ´tolerance factor´, so once a bout is initially detected, the bout is allowed to experience no more than the number of "Drop time" minutes outside of the minimum and maximum count levels. As long as the number of "Drop Time" minutes do not exceed the specified threshold, the bout will continue and will be considered as a single episode. If this number is exceeded, the bout ends,or never begins if the minimum time length has not been encountered.

By using this algorithm, and considering the entire recorded period, we quantified: (i) the number of PA bouts; (ii) the total time spent in the PA bouts; (iii) the average length of the PA bouts; (iv) the maximal (largest) and minimal time (shortest) in PA bouts; (v) the total count level of the PA bouts.

## Physical Activity Levels and Moderate-to-Vigorous Physical Activity (MVPA).

By using the CPM data, PA intensity levels were quantified for a period of time according to the ´Freedson Adult VM3 (2011)´ (Sasaki et al., 2011) and ´Freedson Children (2005)´ cut points, for subjects ≥19and ≤18 y, respectively.

In adults, PA intensity is classified into four cut points based on VM (and counts are based on 60 s epoch):

• Light PA: 0 - 2689 CPM

• Moderate PA: 2690 - 6166 CPM

• Vigorou PA: 6167 - 9642 CPM

• Very Vigorous PA: 9643 - ∞ CPM,

while in children and adolescents (not included in this work) the PA intensityis classified into five cut points:

• Sedentary PA: 0 - 149 CPM

• Light PA: 150 - 499 CPM

• Moderate PA: 500 - 3999 CPM

• Vigorous PA: 4000 - 7599 CPM

• Very Vigorous PA: 7600 - ∞ CPM.

For each PA intensity category (e.g., light PA, moderate PA), we quantified, during the entire recorded period: (i) total time (minutes) and (ii) percentage of time in this category. Finally, we determined the MVPA for each subject, i.e., the amount of time spent by a subject above the "Moderate PA" intensity cut point level, thus indicating "significant” activity (Physical Activity Guidelines for Americans, 2008). The minimum value for MVPA classification was 2690 and 500 CPM for subjects ≥19 and ≤18 y, respectively (based on VM and 60 s epoch). Considering the entire recorded period, we quantified: (i) total amount of MVPA time (minutes), (ii) percentage of MVPA time and (iii) average MVPA time in minutes/day.

## Steps

Wearable activity monitors are commonly used to detect steps, another useful measure to quantify PA. Considering the recorded period, we determined: (i) Total steps counts; (ii) average steps per count, (iii) maximal steps per count, and (iv) Average Steps per minute.

## Sedentary Behavior Analysis: Sedentary Bouts and Breaks.

The Sedentary Behavior analysis identifies bouts of low PA, which allows a better characterization of a subject's sedentary time (e.g., levels, patterns), other than identifying non-wear as sedentary time. A sedentary bout was defined as one or more consecutive minutes with less than 100 CPM, while Sedentary breaks in sedentary time was calculated as any interruption in sedentary time lasting one minute or longer in which the accelerometer CPM rose up toor above 100 (Saunders et al., 2013). By using these criteria and considering the entire recorded period, we quantified the following sedentary bout- and breaks-related indexes: (i) Total number of sedentary bouts and breaks; (ii) Total time (minutes) of sedentary bouts and breaks (sum of all the times between sedentary bouts in the dataset); (iii) Average time (minutes) of sedentary bouts and breaks (an average of all of the times between sedentary bouts); (iv) Maximum (longest) and Minimal (shortest) Time of sedentary bouts and breaks (v) Daily average time (minutes) of sedentary bouts and breaks (total length of sedentary bouts or breaks divided by the total valid days in the dataset).

## References

1. Choi, L., Liu, Z., Matthews, C.E., Buchowski, M.S. (2011). Validation of accelerometer wear and nonwear time classification algorithm. *Med Sci Sports Exerc*. 43(2):357-64. doi: 10.1249/MSS.0b013e3181ed61a3.
2. Edwardson, C.L., Gorely, T. (2010). Epoch length and its effect on physical activity intensity. *Med Sci Sports Exerc.* 42(5):928-34. doi: 10.1249/MSS.0b013e3181c301f5.
3. Freedson, P., Pober, D., Janz, K.F. (2005). Calibration of accelerometer output for children. *Med Sci Sports Exerc.* 37(11 Suppl):S523-30. doi: 10.1249/01.mss.0000185658.28284.ba.
4. Freedson, P.S., Melanson, E., Sirard, J. (1998). Calibration of the Computer Science and Applications, Inc. accelerometer. *Med Sci Sports Exerc.* 30(5):777-81. doi: 10.1097/00005768-199805000-00021.
5. Physical Activity Guidelines for Americans (2008). U.S. Department of Health and Human Services. https://health.gov/sites/default/files/2019-09/paguide.pdf
6. Sasaki, J.E., John, D., Freedson, P.S. (2011). Validation and comparison of ActiGraph activity monitors. *J Sci Med Sport*. 14(5):411-6. doi: 10.1016/j.jsams.2011.04.003.
7. Saunders, T.J., Tremblay, M.S., Mathieu, M.È., Henderson, M., O'Loughlin, J., Tremblay, A., Chaput, J.P.; QUALITY cohort research group (2013). Associations of sedentary behavior, sedentary bouts and breaks in sedentary time with cardiometabolic risk in children with a family history of obesity. *PLoS One*. 8(11):e79143.
8. Trost, S.G., McIver, K.L., Pate, R.R. (2005). Conducting accelerometer-based activity assessments in field-based research. Med Sci Sports Exerc. 37(11 Suppl):S531-43. doi: 10.1249/01.mss.0000185657.86065.98.
